# Studies on the Glutathione-Dependent Formaldehyde-Activating Enzyme from *Paracoccus denitrificans*

**DOI:** 10.1371/journal.pone.0145085

**Published:** 2015-12-16

**Authors:** Richard J. Hopkinson, Ivanhoe K. H. Leung, Tristan J. Smart, Nathan R. Rose, Luc Henry, Timothy D. W. Claridge, Christopher J. Schofield

**Affiliations:** 1 Chemistry Research Laboratory, Department of Chemistry, University of Oxford, Oxford, United Kingdom; 2 School of Chemical Sciences, The University of Auckland, Auckland, New Zealand; Martin-Luther University Halle-Wittenberg, GERMANY

## Abstract

Formaldehyde is a toxin and carcinogen that is both an environmental pollutant and an endogenous metabolite. Formaldehyde metabolism, which is probably essential for all aerobic cells, likely proceeds via multiple mechanisms, including via a glutathione-dependent pathway that is widely conserved in bacteria, plants and animals. However, it is unclear whether the first step in the glutathione-dependent pathway (i.e. formation of *S*-hydroxymethylglutathione (HMG)) is enzyme-catalysed. We report studies on glutathione-dependent formaldehyde-activating enzyme (GFA) from *Paracoccus denitrificans*, which has been proposed to catalyse HMG formation from glutathione and formaldehyde on the basis of studies using NMR exchange spectroscopy (EXSY). Although we were able to replicate the EXSY results, time course experiments unexpectedly imply that GFA does not catalyse HMG formation under standard conditions. However, GFA was observed to bind glutathione using NMR and mass spectrometry. Overall, the results reveal that GFA binds glutathione but does not directly catalyse HMG formation under standard conditions. Thus, it is possible that GFA acts as a glutathione carrier that acts to co-localise glutathione and formaldehyde in a cellular context.

## Introduction

Formaldehyde (HCHO) is produced in cells during redox processes including during the enzymatic demethylation of methylated nucleic acids and proteins [1–3]. Above threshold levels, HCHO is toxic to cells and animals including humans; acute exposure either through inhalation or ingestion may result in convulsions and renal failure, whereas chronic exposure is linked to increased risk of cancers including nasopharyngeal cancer and leukaemia[4,5].

**Fig 1:**
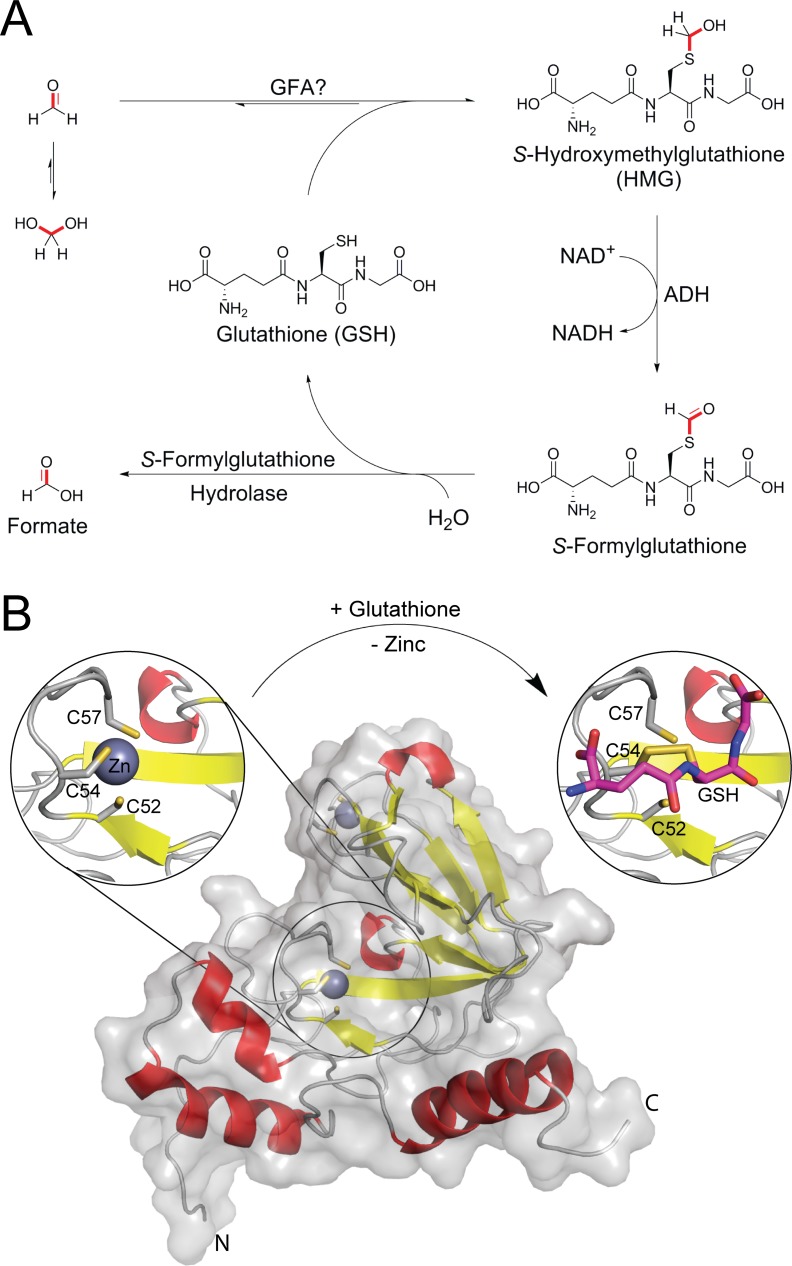
Overview of glutathione-dependent formaldehyde metabolism. (A) Summary scheme of glutathione-dependent formaldehyde metabolism. Formaldehyde (HCHO) reacts with glutathione (GSH) via its nucleophilic thiol group to form *S*-hydroxymethylglutathione (HMG), which is a substrate of glutathione-dependent alcohol dehydrogenase (ADH, ADH5 in humans). The product, *S*-formylglutathione, is then further metabolised by *S*-formylglutathione hydrolase to give formate and GSH. The reaction of HCHO and GSH, i.e. the first step in GSH-dependent metabolism, occurs spontaneously in aqueous solution; however, the reaction might also be catalysed by GFA (and homologues in other organisms, e.g. CENPV in humans[13]). There is also evidence, at least *in vitro*, that GSH can react with HCHO to form cyclised adducts[10–12]. (B) Views of X-ray crystal structures of GFA from *Paracoccus denitrificans* (PDB IDs: 1X6M and 1XA8[14]). The GFA domain contains two zinc binding sites; one zinc ion is coordinated by four cysteinyl thiols (C31, C33, C99 and C102) in a tetrahedral geometry, whereas the other zinc ion is coordinated by three cysteinyl thiols (C52, C54 and C57) in a trigonal planar geometry. Crystallographic studies have proposed that GSH binding induces translocation of the second zinc ion (circles)[14].

A predominant pathway assigned for HCHO metabolism in eukaryotic cells involves its reaction with the tripeptide glutathione (GSH)[6,7]. GSH reacts with HCHO via its nucleophilic thiol group to form the intermediate *S*-hydroxymethylglutathione (HMG). HMG may then be oxidised by an alcohol dehydrogenase (ADH5 in humans) to form *S*-formylglutathione, which is then further metabolised by *S*-formylglutathione hydrolase to give formate, thus returning GSH to the cellular pool (Fig 1A). Although GSH-dependent HCHO metabolism likely occurs in most, if not all, human cells (based on the abundance of GSH and expression of the relevant detoxifying enzymes)[6] there are currently few kinetic studies of this pathway in cells. However, recent work has indicated that the presence of the GSH-dependent HCHO metabolism pathway is important for tolerating HCHO-induced toxicity in cells and animals deficient in the Fanconi Anemia DNA repair pathway[8,9]. Studies in aqueous solution suggest that the non-enzymatic reactions of GSH and HCHO are dynamic and complex, and can result in the formation of multiple cyclic GSH-HCHO adducts[10–12]. It is unclear whether formation of such adducts, as well as HMG, are involved in HCHO metabolism, or whether HMG formation is enzyme-catalysed. It is also unclear whether the GSH-dependent HCHO metabolism pathway predominates in all human cell types and cellular compartments, e.g. the nucleus or cytoplasm.

Recent work with *Paracoccus denitrificans* using NMR analyses employing exchange spectroscopy (EXSY) resulted in the identification of a novel GSH-utilising HCHO-detoxifying enzyme[13]. This protein, named glutathione-dependent formaldehyde-activating enzyme (GFA), is proposed to accelerate the reaction of GSH and HCHO to form HMG, the first step in GSH-dependent HCHO metabolism. Crystallographic studies have indicated that GFA contains two zinc ions coordinated via four and three cysteine residues respectively, with the latter adopting a trigonal planar geometry. GFA catalysis is proposed to proceed via a highly unusual mechanism involving translocation of this second zinc ion to another undefined site on the protein. HMG formation is then catalysed at this site before the zinc ion returns to the trigonal planar site after catalysis (Fig 1B and S1 Scheme)[14].

A human homologue of GFA, centromere-associated protein V (CENPV, for sequence alignment see S1 Fig), is required for efficient centromere formation and maintenance; elevated levels of CENPV in HeLa cells result in aberrant condensation of pericentromeric heterochromatin that is dependent upon the presence of the trigonal planar zinc binding site[15]. CENPV depletion also induces rapid cell death. Collectively, these studies suggest that GFA and its homologues in higher animals may play important roles in HCHO metabolism in a manner relating to chromatin function.

We are interested in the fate and roles of HCHO produced by *N*-demethylation reactions of methylated nucleic acid bases and protein components of chromatin. Following on from pioneering work in the 1970s[16], more recent studies have revealed that demethylation of *N*-methylated histone (and other) proteins as well as methylated nucleic acids to give the demethylated product and HCHO is common in eukaryotes. In humans, protein *N*-demethylation reactions producing HCHO are reported to be catalysed by members of the amine oxidase and the 2-oxoglutarate, oxygen and iron(II)-dependent oxygenase superfamilies (for reviews see [3,17]). Both enzyme families catalyse the oxidation of *N*-methyl groups; the amine oxidases (KDM1A-B in humans) facilitate hydride transfer from the methyl group onto a flavin adenine dinucleotide cosubstrate (amine oxidases[18]), whereas 2-oxoglutarate- and iron(II)-dependent oxygenases (ABH2/3/5, FTO, KDM2-7 in humans) catalyse *N*-methyl hydroxylation with concomitant conversion of 2-oxoglutarate and oxygen to succinate and carbon dioxide[1,19].

Here, we report studies on the reaction of GSH and HCHO in the presence of recombinant GFA *in vitro* and in cell lysate from *Escherichia coli* (*E*. *coli*). Although we were able to replicate findings from the previously reported EXSY experiments (i.e. increased EXSY cross-peak intensities between GSH and HMG upon addition of GFA[13]), ^1^H NMR time-course experiments did not reveal GFA-catalysed acceleration of the formation of HMG. Further, addition of recombinant GFA to cell lysate did not appear to affect HCHO metabolism; however, GFA was observed to bind GSH by NMR and mass spectrometric binding experiments. Overall, although our findings indicate that GSH can bind to GFA, they imply that recombinant GFA does not catalyse HMG formation from GSH and HCHO in any detectable way under standard aqueous conditions.

## Materials and Methods

### Materials

Deuterated Tris buffer was from Cortecnet (France). γ-Glutamyl-serinyl-glycine and γ-glutamyl-D-cysteinyl-glycine were from Peptide Synthetics (U. K.). All other reagents were from The Sigma-Aldrich Chemical Company. HCHO solution was prepared by heating paraformaldehyde powder in H_2_O in a glass vial using a heat gun at >100°C until the powder had dissolved. Pre-prepared formalin solution was not used so as to avoid contamination from methanol. [^13^C]-labelled HCHO ([^13^C]-HCHO) was purchased as 20 wt % in H_2_O from Sigma-Aldrich.

### Protein Production

Recombinant wild-type GFA was expressed from its gene cloned in a modified pET16b plasmid (to encode for recombinant protein with a Tobacco Etch Virus nuclear inclusion-a endopeptidase (TEV protease) cleavage site between the His-Tag and the insert, S. Becker, Göttingen) in *E*. *coli* BL21 (DE3) cells grown on 2 × Tryptone Yeast (2TY) Extract medium[20–22]. The protein was purified via Ni-affinity before removal of the *N*-terminal His-Tag using TEV protease[23] and size-exclusion chromatography. Some experiments were carried out using His-Tagged GFA (unless stated, GFA (without His-tag) was used). Aliquots were stored in 50 mM Tris buffer in H_2_O pH 7.5 (His-Tagged GFA was stored in 20 mM HEPES buffer in H_2_O pH 7.5). The GFA C54A variant was generated using the QuickChange site-directed mutagenesis kit (Stratagene) and the mutation was confirmed by DNA sequencing. This plasmid was then transformed into *E*. *coli* BL21 (DE3) and expressed and purified as described above (without His-Tag, stored in 50 mM Tris buffer in H_2_O pH 7.5).

### NMR Analyses

NMR experiments were carried out using a Bruker Avance III 700 MHz spectrometer equipped with an inverse TCI cryoprobe optimised for ^1^H observation and installed with Topspin 3 software. Each sample was prepared in a microcentrifuge tube (either 75 μL or 160 μL final volume) before being transferred to either a 2 mm or a 3 mm Bruker MATCH NMR tube (Hilgenberg), centrifuged for a few seconds using a hand centrifuge, and transferred to the spectrometer for NMR analysis. Where applicable, mixtures were left in microcentrifuge tubes to reach equilibrium before transfer to NMR tubes (15–30 min). All experiments were carried out at 298 K unless otherwise stated.

For ^1^H NMR experiments, the solvent resonance was removed by excitation sculpting using a 2 ms 180^°^ sinc pulse[24]. Time-course experiments were conducted using automated routines; 20 analyses were performed on each sample, each accumulating 16 transients corresponding to 89 s (or 80 s for the experiments with lysate) of total measurement time.

EXSY analyses were performed using 1D NOESY pulse sequences using selective refocusing with Gaussian pulses[13,25,26]. 2D experiments were run accumulating 16 transients with a mixing time (τ_m_) of 400 ms. 1D experiments were performed accumulating 64 transients with τ_m_ of 32–400 ms (for comparative studies, experiments were run with a τ_m_ of 80 ms). For 1D EXSY analyses, the resonance at δ_H_ 2.95 ppm (corresponding to one β-cysteinyl proton of HMG) was selectively irradiated.

Saturation transfer difference (STD) analyses were performed using the Bruker pulse sequence, in which selective saturation of the protein resonance was achieved by a train of Gaussian-shaped pulses (50 ms)[27]. A 2 ms sinc pulse was employed for excitation sculpting water suppression. The on-resonance irradiation was selected at 593 Hz and the off-resonance at 35000 Hz. The total saturation time and relaxation delays were 10 s and 18 s respectively.

Water-ligand observed via gradient spectroscopy (waterLOGSY) analyses were performed using the pulse sequence described by Dalvit *et al*[28]. Solvent water excitation was achieved using a 16 ms selective rectangular shape pulse set at the H_2_O frequency. The relaxation delay was 2 s and the τ_m_ was 1 s. A 2 ms sinc pulse was used for excitation sculpting water suppression. Each experiment accumulated 64–256 transients at 280 K.

Unless otherwise stated, all errors are reported as standard deviations of the mean (n = 3).

### Cell Lysate Experiments

For work with cell lysates, untransformed *E*. *coli* BL21 (DE3) cells were grown in 2TY medium at 37°C for 16 hours under aerobic conditions (to stationary phase) before centrifugation and re-suspension in 50 mM Tris buffer pH 7.5 (0.5 mg of cells per mL). The cells were lysed by sonication (20 × 2 s) and the supernatant separated from insoluble particulates by centrifugation and then filtration using a 0.45 μm syringe filter (Sartorius Stedim Biotech). NMR experiments (160 μL total volume) were then conducted on the supernatant after addition of [^13^C]-HCHO and D_2_O (16 μL).

## Results

**Fig 2:**
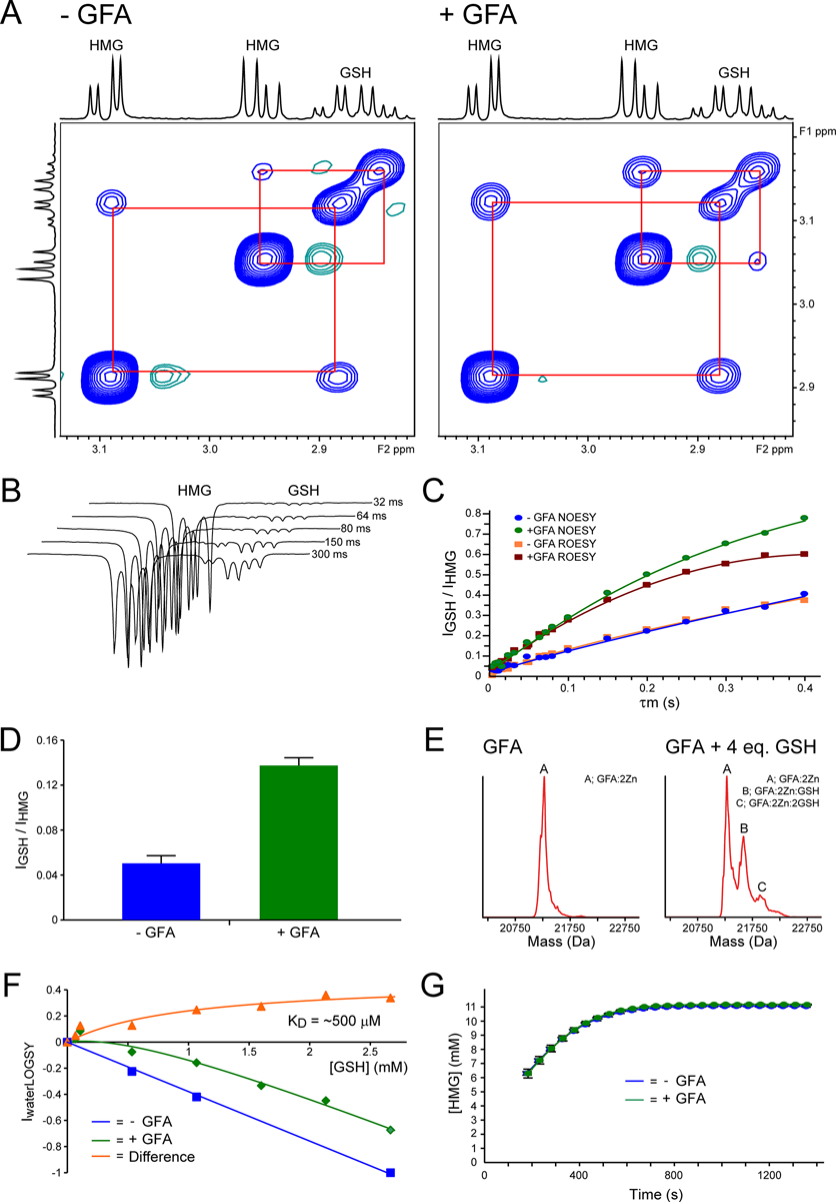
GFA is a GSH-binding protein that induces an increase in EXSY correlation intensities between GSH and HMG, but does not catalyse HMG formation / fragmentation. (A) 2D EXSY spectra of equilibrium mixtures of GSH (initial concentration 15 mM) and HMG in the absence (left) and presence (right) of GFA (20 μM). Mixing time (τ_m_) = 400 ms. EXSY-correlation intensities between GSH and HMG are increased in the presence of GFA. (B) 1D EXSY spectra of equilibrium mixtures of GSH (initial concentration 15 mM) and HMG in the presence of GFA (20 μM) conducted at different mixing times (τ_m_ = 32–300 ms). Irradiation (inversion) of a β-cysteinyl resonance of HMG (δ_H_ 2.95 ppm) induced an exchange correlation at δ_H_ 2.87 ppm, corresponding to the β-cysteinyl resonance of GSH, which increased in intensity at longer mixing times. (C) Graph showing the intensity of the GSH cross-peak relative to the inverted HMG resonance in the absence and presence of GFA at different mixing times, using either NOESY or ROESY pulse sequences. τ_m_ = 4–400 ms. (D) Bar graph showing the intensity of the GSH 1D EXSY-correlation relative to the irradiated HMG resonance in the absence (blue) and presence (green) of GFA (τ_m_ = 80 ms). The build-up rates of the 1D EXSY analyses (note: a τ_m_ of 80 ms is within the linear range of the EXSY build-up curves, Fig 2C) correlate with the rates of GSH/HMG exchange at equilibrium. Therefore, the observed increase in correlation intensity in the presence of GFA implies an increase in GSH/HMG inter-conversion rate. (E) Non-denaturing MS analyses of GSH binding to GFA. Two new peaks corresponding to the masses of monomeric GFA (with two zinc ions in complex) bound to one and two GSH molecules respectively were observed upon incubation with GSH (4 equivalents, right). (F) Binding curve of GSH binding to GFA obtained using waterLOGSY. Selective irradiation of the solvent H_2_O ^1^H resonance results in magnetisation transfer to GSH, resulting in the emergence of GSH ^1^H resonances with opposite sign to the irradiated H_2_O resonance. The (negative) intensities of the GSH resonances are linearly dependent on the GSH concentration (blue). Addition of GFA results in a slower net tumbling rate for GSH in solution due to binding with GFA. The slower tumbling rate leads to ‘(more) positive’ GSH resonance intensities as a function of the extent of ligand binding (green). Subtraction of the intensities in the absence (blue) and presence (green) of GFA gives a normalised binding curve (orange, K_D_ value of roughly 500 μM assuming binding of one GSH molecule per GFA subunit). The experiments were carried out at 280 K. τ_m_ = 1 s. (G) Graph showing production of HMG from mixtures of GSH (13.3 mM) and HCHO (13.3 mM) in the absence (blue) and presence (green) of GFA (16 μM) in BisTris buffer pH 6.0. GFA does not affect the initial HMG formation rate.

In order to investigate the effect(s) of GFA on the reactions of GSH and HCHO in aqueous solution, NMR studies were carried out using recombinant GFA purified from *E*. *coli* (>95% pure by SDS-PAGE, S2–S4 Figs)[13,14]. Our initial experiments focussed on reproducing the previously observed increases in EXSY-correlation intensities between GSH and HMG in the presence of GFA[13]. EXSY, an NMR-based method, utilises the same pulse sequences applied to detect nuclear Overhauser effect (nOe) correlations between spatially close nuclei; EXSY-correlations (cross-peaks) are classically observed between two species, with distinct chemical shifts, that exist in a slow exchange equilibrium (e.g. chemical or conformational). The initial growth ratios of the EXSY-correlations are proportional to the rate of exchange between the species, and therefore, EXSY may be used as a quantitative measure of equilibrium dynamics, e.g. HCHO/HMG inter-conversion[13]. Mixtures of GSH (15 mM) and HCHO (15 mM) were prepared in deuterated Tris buffer in 90% H_2_O / 10% D_2_O pH* 7.5 and were left for 30 minutes to reach equilibrium (note: evidence of a previously characterised Tris-HCHO adduct[29] was observed but was at low levels relative to free HCHO) before addition of either GFA (20 μM final concentration) or buffer (as a control) prior to 2D EXSY analysis. As reported[13], reproducible EXSY correlations between resonances corresponding to the β-cysteinyl protons of GSH and HMG increased in intensity in the sample containing GFA (with both poly-histidine (His)-Tagged and non-tagged GFA batches), relative to the control sample in the absence of GFA (Fig 2A). Similar findings were also observed in 1D EXSY experiments, where the HMG β-cysteinyl resonance at δ_H_ 2.95 ppm was selectively irradiated (an increase of roughly 2 to 3-fold, Fig 2B, 2C and 2D). 1D EXSY analyses were also carried out using a rotating-frame NOESY pulse sequence (ROESY), which revealed identical build-up curves of the intensities of the GSH/HMG cross-peaks at different mixing times to those observed using the NOESY pulse sequence (Fig 2C; for interpretation of the ROESY analyses, see Discussion); no increase in the EXSY-correlation intensity (relative to no-enzyme control) was observed upon incubation with a GFA variant (GFA C54A, S5 Fig), which contains a compromised zinc-binding site, as shown by non-denaturing electrospray ionisation mass spectrometry (S6 Fig). Further, EXSY analysis after incubation of the GFA-containing samples in boiling water to denature the protein for two minutes resulted in a decrease in cross-peak intensities (S5 Fig). Addition of zinc ions (20 μM) to the reaction mixtures did not affect the EXSY cross-peak intensities, either in the absence or presence of GFA (note: purified recombinant GFA contains two zinc binding sites[14]). The ratio of EXSY correlation intensities between the samples with and without GFA was found to be pH-dependent: at low pH (pH* 5.5), the ratio was most significant, although the respective intensities were smaller than at higher pH values (S7 Fig). These results suggest a predominance of protein-independent GSH/HMG inter-conversion pathways at higher pH, presumably due to the increased nucleophilicity of the GSH thiolate[12]. Incubation with GFA was also observed to increase cross-peak intensities between GSH and both *S*-hydroxyethylglutathione (HEG, from reaction of GSH with acetaldehyde, S8 Fig) and *S*-hydroxypropylglutathione (HPG, from reaction of GSH and propionaldehyde, S8 Fig) respectively (for scheme, see S2 Scheme). Overall, these NMR analyses reveal that GFA induces an increase in EXSY cross-peak intensities between GSH and hydroxyalkyl-GSH adducts derived from reactions not only with HCHO, but also with acetaldehyde and propionaldehyde, implying binding of GSH / the hydroxyalkyl-GSH adducts to GFA induces an increase in their inter-conversion rates.

The binding of GFA to GSH was then investigated. Binding analyses were carried out using two ligand-observed NMR-based methods (saturation transfer difference spectroscopy (STD), and water-ligand observed via gradient spectroscopy (waterLOGSY)) and non-denaturing mass spectrometry. STD and waterLOGSY are NMR methods which monitor the transfer of magnetisation from a selectively irradiated species to a ligand when the two are close in space[27,30]. In the case of STD, ^1^H nuclei on the protein of interest are irradiated; magnetisation transfer from the protein to the ligand occurs when the ligand is bound to the protein, and may be used as a measure of ligand binding providing the ligand experiences rapid on-off exchange with the protein over the time course of the experiment. In waterLOGSY, magnetisation transfer is observed between the ligand and irradiated solvent H_2_O; when the small molecule ligand population is free in solution (i.e. not bound to protein), magnetisation transfer results in signals for the ligand with opposite sign to the irradiated H_2_O ^1^H resonance, due to the fast tumbling rate of the ligand during the experiment[30]. When the ligand is bound to protein, the net tumbling rate is decreased, which results in perturbation of the signal intensity. Therefore, monitoring the net difference in ligand signal intensities in the presence and absence of protein provides evidence for ligand protein binding. In each of the STD, waterLOGSY and MS analyses, GSH was observed to bind to GFA, with an estimated dissociation constant (K_D_) of 500 μM using waterLOGSY (Figs 2E and 2F and S9 and S10). Further, GSH was not observed to bind the GFA C54A variant using waterLOGSY (S11 Fig). Interestingly, GFA was not observed to bind GSH analogues including *N*-acetyl-cysteine, γ-glutamyl-serinyl-glycine, oxidised glutathione (GSSG) and *S*-methyl-glutathione using waterLOGSY (S12 Fig). GFA therefore has at least some selectivity for GSH binding. 1D EXSY analyses on mixtures containing HCHO and *N*-acetyl-cysteine, γ-glutamyl-cysteine, *N*-acetyl-cysteamine and γ-glutamyl-D-cysteinyl-glycine (D-GSH) respectively, did not reveal increased correlation intensities upon addition of GFA (S13 Fig), suggesting the effect of GFA on mixtures is dependent on GSH (and/or possibly HMG) binding to GFA (note: although HMG binding was not observed in the MS experiments, it is possible that HMG fragments under the ionisation conditions). Under our conditions, binding of HCHO to GFA was not observed using [^13^C]-NMR experiments (via monitoring the intensity of the [^13^C]-resonance of [^13^C]-labelled-HCHO upon addition of GFA) or using non-denaturing mass spectrometry (within limits of detection). ^1^H NMR and non-denaturing MS experiments also did not reveal binding of either acetaldehyde or propionaldehyde to GFA. Finally, HMG, HEG and HPG were not observed to bind GFA in non-denaturing MS experiments, although it is possible that the adducts fragment under the ionisation conditions.

We then investigated whether GFA accelerates the initial rate of HMG formation. Assuming GFA accelerates the rates of both HMG formation and fragmentation (to give GSH and HCHO), the time required for the GSH + HCHO / HMG equilibrium to be established (from mixing of GSH and HCHO) should be accelerated upon addition of GFA, thus resulting in an increase in the initial HMG formation rate. Reaction mixtures of GSH and HCHO were prepared in deuterated Tris buffer as described above, and the samples (either with or without GFA) were monitored over early time-points using ^1^H NMR. Interestingly, no detectable differences in initial HMG formation rates were identified in the samples (Fig 2G)–the experiments were attempted on multiple (>10) occasions with different protein batches, including with both His-tagged and non-tagged GFA (S14 Fig). The initial formation rates of HEG (from GSH and acetaldehyde) and HPG (from GSH and propionaldehyde) could not be determined as the reaction mixtures had already reached equilibrium before the first NMR experiment.

**Fig 3:**
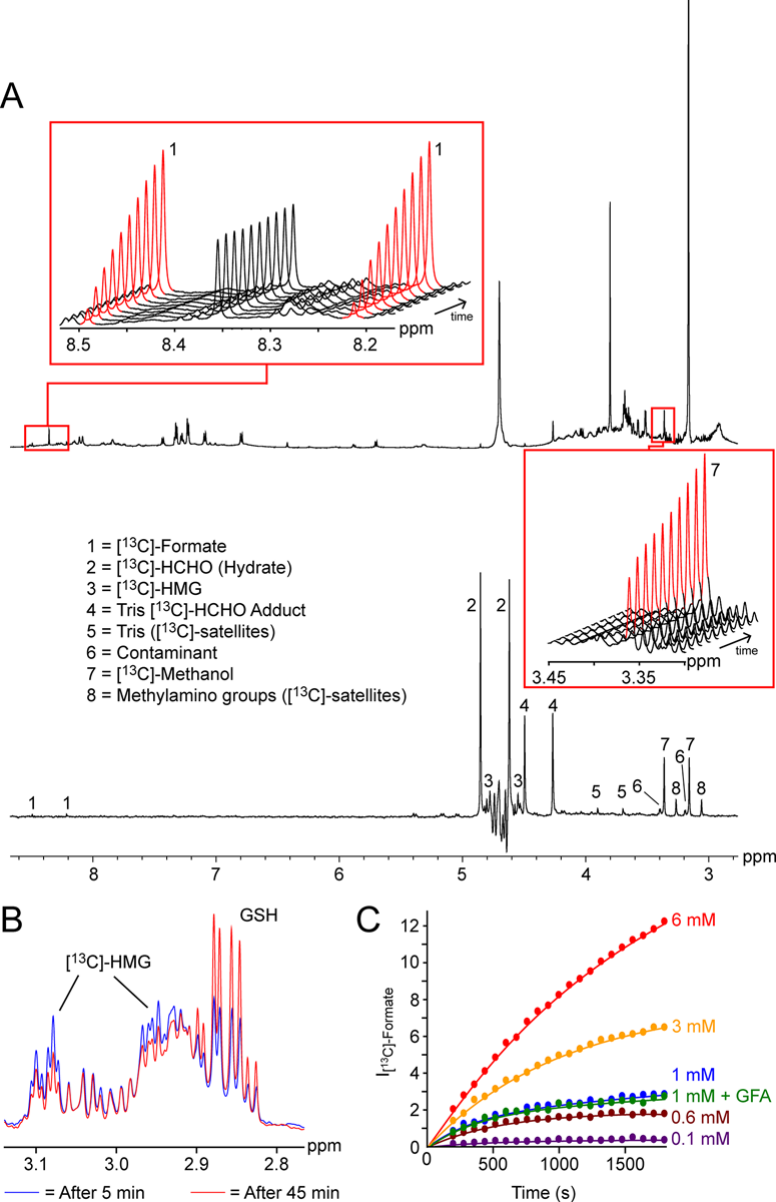
GFA does not affect HCHO metabolism in *E*. *coli* cell lysate. (A) ^1^H NMR (top) and 1D-^13^C-HSQC spectra (bottom) of *E*. *coli* BL21 (DE3) cell lysate (0.5 mg/mL in 50 mM Tris buffer pH 7.5) incubated with [^13^C]-HCHO (6 mM). Resonances in the 1D-HSQC spectra are annotated. [^13^C]-satellites for the Tris buffer are observed in the 1D-HSQC spectrum due to its high abundance of in the mixture. The doublet resonance at δ_H_ 3.25 ppm is assigned to methylamino groups from the lysate. Inset top left and bottom right: ^1^H NMR spectra of *E*. *coli* BL21 (DE3) cell lysate (0.5 mg/mL in 50 mM Tris buffer pH 7.5) incubated with [^13^C]-HCHO (6 mM) over time. Production of ^13^C-formate (as indicated by the increase in intensity of the doublet resonance at δ_H_ 8.36 ppm, red top left) and [^13^C]-methanol (as indicated by the increase in intensity of the resonance at δ_H_ 3.36 ppm, red bottom right) are clearly observed. Only one half of the expected doublet resonance is observed for [^13^C]-methanol in the ^1^H spectra due to overlap with the Tris buffer. Note: the relatively high initial [^13^C]-methanol level (relative to the level of [^13^C]-formate) is due to contamination of the commercial source of [^13^C]-HCHO (S15 Fig). (B) ^1^H NMR spectra of *E*. *coli* BL21 (DE3) cell lysate (0.5 mg/mL in 50 mM Tris buffer pH 7.5) incubated with GSH (4 mM) and [^13^C]-HCHO (6 mM) after 5 min (blue) and 45 min (red) respectively. [^13^C]-HMG (as indicated by the resonances at δ_H_ 2.96 ppm and δ_H_ 3.09 ppm) was most abundant over early time points and decreased during the experiment, which correlated with time-dependent formation of [^13^C]-formate, [^13^C]-methanol and GSH. Resonances corresponding to [^13^C]-HMG were poorly resolved in the time-course experiments without added GSH due to overlap with other resonances (see S16 Fig). (C) GFA does not affect the rate of formate production in *E*. *coli* cell lysate. Initial [^13^C]-formate production rate is dependent on the concentration of added [^13^C]-HCHO over the tested range; however, addition of recombinant GFA (20 μM) to the lysate before incubation with 1 mM [^13^C]-HCHO (green) does not affect the rate of [^13^C]-formate production (relative to the control without added enzyme, blue).

The results from the *in vitro* experiments prompted studies using *E*. *coli* cell lysates as a more biologically-relevant model system. *E*. *coli* cells are reported to utilise multiple HCHO metabolism pathways including the GSH-dependent pathway[31,32]; however, GFA is not produced. *E*. *coli* BL21 (DE3) cells were grown in 2TY medium and were lysed by sonication in 50 mM deuterated Tris buffer in H_2_O pH 7.5 (0.5 mg/mL). [^13^C]-labelled HCHO (6 mM, for a reference ^1^H NMR spectrum of the commercial batch of [^13^C]-HCHO, see S15 Fig) was then added to the lysates and the mixture was analysed using ^1^H NMR and 1D-^1^H-^13^C-HSQC NMR to accentuate the ^1^H-resonances attached to [^13^C] labels (i.e. ^1^H-resonances derived from metabolites / reaction products of [^13^C]-HCHO). Three predominant products of reactions with [^13^C]-HCHO were identified, which were assigned as [^13^C]-labelled formate, [^13^C]-labelled methanol and HMG labelled at the hydroxymethyl carbon ([^13^C]-HMG, Fig 3A). In *E*. *coli*, methanol and formate may be produced from HCHO via direct reduction or oxidation of HCHO respectively, reactions which are coupled to oxidation/reduction of NAD^+^/NADH. Alternatively, formate can be produced via the GSH-dependent pathway via HMG (S3 Scheme)[31,32]. It is also possible that other as yet undefined pathways of formate and methanol formation from HCHO exist in *E*. *coli*.

The concentration of [^13^C]-HMG in the samples, formed via reaction of [^13^C]-HCHO with GSH, was highest at early time-points, decreasing slowly over the course of the experiment (Figs 3B and S16). Importantly, the rates of both [^13^C]-methanol and [^13^C]-formate production were dependent upon the concentration of added [^13^C]-HCHO (Fig 3C, production of [^13^C]-methanol was only observed in the sample with 6 mM [^13^C]-HCHO). These findings suggest that, at low [^13^C]-HCHO concentrations, at least some pathways of [^13^C]-formate (and [^13^C]-methanol) production are limited by the availability of either free [^13^C]-HCHO or [^13^C]-HMG. Hence, assuming GFA catalyses the GSH/HMG inter-conversion, addition of recombinant GFA to the lysates should increase the dynamicity of the GSH + HCHO / HMG equilibrium, allowing the equilibrium to respond more quickly to the sequestering of free [^13^C]-HCHO and/or [^13^C]-HMG to form [^13^C]-methanol and [^13^C]-formate. Therefore, assuming that the rates of the GSH-dependent and non-GSH-dependent HCHO metabolism pathways are different in the lysates, (note: the [^13^C]-HCHO signal (hydrated form) could not be analysed due to suppression of the nearby water resonance), addition of GFA should induce an increase in formate and methanol formation rates as a consequence of increasing the GSH/HMG inter-conversion rate. However, either with or without added GSH, the presence of GFA in the lysate (added in recombinant form) did not affect the formation rates of [^13^C]-formate upon addition of [^13^C]-HCHO (Fig 3C, at 1 mM, [^13^C]-methanol production was not observed). Overall, the lysate experiments do not support a proposed role for GFA catalysis in HMG formation or degradation.

## Discussion

GFA was initially identified as a GSH-dependent HCHO-metabolising enzyme by 2D EXSY analyses and shown to be a zinc-binding enzyme by crystallographic studies[13,14]. In our work, binding experiments using both NMR and mass spectrometric techniques revealed that GFA binds GSH; however, although we observed that GFA can induce increases in EXSY cross-peak intensities between the β-cysteinyl protons of GSH and HMG, our studies demonstrate that GFA does not increase the rate of reaction of GSH and HCHO to form HMG, at least under the tested conditions. The biological relevance of these findings are supported by experiments using cell lysates, which did not reveal an acceleration of HCHO metabolism (by a GSH-dependent pathway or any other pathway) upon addition of GFA. Overall, the evidence suggests that GFA is a GSH-binding protein but does not catalyse HMG formation under standard conditions; although roles in HCHO metabolism cannot be ruled out, any effects would either likely not involve catalysis of HMG formation, or may be restricted to situations / environments where conditions are perturbed. Such assignments require further validation.

The conclusion that changes in EXSY cross-peak intensities induced by GFA are not indicative of GFA-catalysed HMG formation and fragmentation raises the question of how the observed changes arise and of their significance. Typically, EXSY experiments involving small molecules have been applied to study chemical transformations, such as conformational interconversions or ligand exchange processes[26]. In these cases, the mechanism of magnetisation ‘transfer’ involves the physical interconversion of species that exist at equilibrium, and the observed correlation intensities are dependent on the rate(s) of exchange between these species. Given that GFA binds GSH (with a K_D_ value significantly lower than the GSH concentrations used in the EXSY experiments), and that both denatured GFA and a GFA variant containing a compromised zinc binding site do not induce increased EXSY cross-peak intensities, it seems likely that the observed magnetisation transfer between GSH and HMG is dependent on GFA-GSH binding. One possible exchange pathway may therefore involve binding of both GSH and HMG simultaneously to the GFA active-site; in this case, inter-conversion between the enzyme-bound GSH and HMG by transfer of the hydroxymethyl group may be, in effect, ‘catalysed’ by GFA, although the GSH/HMG populations would remain constant after inter-conversion. It is also possible that GSH and HMG are bound within separate ‘active-sites’ of the GFA homodimer (S17 Fig), which appear sufficiently close to allow transfer of HCHO between the two molecules (S18 Fig).

Another plausible explanation for the observed increase in EXSY correlation intensities upon addition of GFA is that magnetisation may be transferred from GSH/HMG to GFA via nOe spin-diffusion processes, followed by re-magnetisation of another molecule of HMG/GSH binding subsequently at the same site. Such processes are utilised in the interligand nOes for pharmacophore mapping (INPHARMA) method for the screening of small-molecule ligands binding to the same protein active site[33]. In this process, NOESY experiments are used to detect NOEs between the two protein ligands that displace one another in the active-site; a process of potential relevance here since the EXSY sequence is essentially identical to that of NOESY, suggesting the INPHARMA process may contribute to EXSY peak intensities. However, both in the absence and presence of GFA, initial build-up curves of the intensities of the GSH/HMG cross-peaks at different mixing times were identical using NOESY and ROESY pulse sequences (**Fig 2C**); rotating frame nOes (rOes) are of opposite sign to classical nOes observed for large molecules (such as a GFA-GSH complex) and to EXSY exchange peaks, so the presence of these should act to reduce EXSY cross-peak intensities. These findings suggest that NOE spin-diffusion processes do not contribute to the appearance of exchange cross-peaks between GSH and HMG. Overall our data suggest that the increased EXSY peak intensities are due to binding of GSH/HMG to GFA, possibly due to their mutual inter-conversion in the enzyme bound state.

## Conclusions

Cellular studies have provided evidence that both GFA and its human homologue CENPV are involved in formaldehyde metabolism. The combined biochemical and structural results, however, imply that GFA does not catalyse the production of HMG from GSH and HCHO, either in isolated protein form or in crude bacterial cell lysates. However, GFA does bind GSH; thus, one possibility is that GFA / CENPV acts as a ‘store’ for GSH. Given the established roles of targeting domains in enzymes involved in eukaryotic epigenetic regulation, including for histone demethylases, it is possible that GSH and HCHO are ‘channelled’ to one another in a process involving CENPV / GFA. We propose that such channelling is likely to be especially important in the case of the toxic and uniquely reactive carbonyl compound HCHO.

## Supporting Information

S1 Fig Alignment of GFA (residues 23–107) from *Paracoccus denitrificans* and its human homologue CENPV (residues 141–221). Cysteine residues predicted to coordinate zinc ions are highlighted in red (for the tetrahedral coordinated zinc ion) and green (for the trigonal planar coordinated zinc ion) respectively. In this work, full-length GFA was used (GFA WT, residues 1–194); an additional glycine residue and a histidine residue were also present on the *N*-terminus of this construct as a result of TEV protease-mediated removal of the *N*-terminal His-Tag after purification.
(TIF)

S2 Fig SDS-PAGE gels of wild type non-tagged GFA, the GFA C54A variant (non-tagged), and of wild type His-tagged GFA. (TIF)

S3 Fig Denaturing mass spectra of wild-type non-tagged GFA (GFA WT, bottom) and GFA C54A variant (top). Masses are given for each protein.
(TIF)

S4 Fig Circular dichroism spectrum of GFA. (TIF)

S5 Fig Denatured GFA and the GFA C54A variant do not induce an increase in EXSY correlation intensities between GSH and HMG. (A) 1D EXSY spectra of equilibrium mixtures of GSH (initial concentration 15 mM) and HMG in the absence (blue) and presence of GFA (20 μM, green), in the presence of GFA after denaturation (red), and in the presence of GFA C54A (20 μM, yellow). The EXSY-type correlation at δ_H_ 2.87 ppm, corresponding to the β-cysteinyl resonance of GSH, was observed to increase only in the presence of GFA (green), relative to the no enzyme control (blue). (B) Bar graph showing the intensity of the GSH EXSY-correlation relative to the inverted HMG resonance in the presence of (red) GFA after denaturation, and in the presence of (yellow) GFA C54A. Values for samples with and without wild-type GFA are indicated by dashed lines (blue and green respectively, Fig 2C). Denaturation was performed by incubating the protein in boiling water for two minutes. EXSY mixing time (τ_m_) = 80 ms.
(TIF)

S6 Fig Non-denaturing MS spectrum of GFA C54A variant. The major species (A) has a mass of 21184 Da, which is assigned to the protein in complex with one zinc ion (predicted at 21191 Da assuming deprotonation of two zinc-binding residues). The predicted mass of the protein in complex with two zinc ions is 21254 Da (assuming deperotonation of four zinc-binding residues), which is not observed.
(TIF)

S7 Fig Bar graph showing the intensity of the GSH EXSY-correlation relative to the inverted HMG resonance in the absence (blue) and presence (green) of GFA at different pH values. Experiments were conducted in either 50 mM Tris buffer or 50 mM BisTris buffer in D_2_O. GSH (25 μL of 40 mM stock in the one of the above buffers), HCHO (25 μL of 40 mM stock in D_2_O) and D_2_O (20 μL) were left to pre-equilibrate before addition of His-tagged GFA (5 μL of 5.7 mg/mL in 20 mM HEPES in H_2_O pH 7.5) and 1D EXSY analysis. τ_m_ = 80 ms. Inset: Graph showing the ratio of the GSH EXSY-correlation intensities in the absence and presence of GFA at different pH values. Although the EXSY correlation intensities were greater at high pH, GFA induced a greater intensity change (relative to the no enzyme control) at low pH. Errors are reported as standard deviations of the mean (n = 3, except for pH* 6.5 without GFA, where n = 2).
(TIF)

S8 Fig GFA induces increases in EXSY cross-peak intensities in dynamic reaction mixtures of GSH with both acetaldehyde and propionaldehyde. Top: ^1^H NMR spectra showing the formation of GSH-acetaldehyde adducts (*S*-hydroxyethylglutathione, HEG, left) and GSH-propionaldehyde adducts (*S*-hydroxypropylglutathione, HPG, right). Two diastereoisomers of each adduct are observed due to the formation of a stereogenic hemithioacetal group. Bottom: Bar graphs showing the intensities of the GSH EXSY-correlations relative to the inverted HEG resonance (left) and HPG resonance (right) in the absence (blue) and presence (green) of GFA (His-Tagged in 20 mM HEPES buffer pH 7.5, 5 μM). Experiments were conducted in 50 mM BisTris buffer pH* 6.0 in D_2_O, and contained GSH (13.3 mM) and either acetaldehyde (26.7 mM) or propionaldehyde (13.3 mM). τ_m_ = 80 ms. Errors are reported as standard deviations of the mean (for samples with HEG, n = 3; for samples with HPG, n = 2).
(TIF)

S9 Fig STD NMR spectra of a sample containing GSH (13.3 mM) and GFA (36 μM). Spectra with selective irradiation at 593 Hz (corresponding to protein ^1^H resonances, middle) and 35000 Hz (corresponding to no ^1^H resonances, bottom) were collected and then subtracted to give the difference spectrum (top). The observation of ^1^H resonances for GSH in the difference spectrum indicates magnetisation transfer from the irradiated protein to enzyme-bound GSH (in the spectrum with irradiation at 593 Hz). The sample contained His-tagged GFA (10 μL of 5.7 mg/mL stock in 20 mM HEPES buffer in H_2_O pH 7.5), GSH (25 μL of 40 mM stock in Tris buffer in H_2_O pH 7.5) and D_2_O (40 μL).
(TIF)

S10 Fig STD NMR spectra of a sample containing GSH (500 μM) and GFA (36 μM). ^1^H resonances for GSH are observed in the difference spectrum (bottom, see S11 Fig legend for description of how the difference spectrum is obtained). The spectrum with irradiation at 35000 Hz (i.e. no protein irradiation, red), showing the GSH resonances is also shown. The sample contained His-tagged GFA (10 μL of 5.7 mg/mL stock in 20 mM HEPES buffer in H_2_O pH 7.5), GSH (25 μL of 1.5 mM stock in Tris buffer in H_2_O pH 7.5) and D_2_O (40 μL).
(TIF)

S11 Fig waterLOGSY spectra of GSH (533 μM) in the absence (blue) and presence (yellow) of GFA C54A variant (16 μM). The sample contained GSH (5 μL of 8 mM stock in 50 mM Tris buffer in H_2_O pH 7.5), either GFA C54A (5 μL of 5.0 mg/mL in 50 mM Tris buffer in H_2_O pH 7.5) or 50 mM Tris buffer in H_2_O pH 7.5 (5 μL), H_2_O (61.25 μL) and D_2_O (3.75 μL). Experiments were carried out at 280 K. t_m_ = 1 s.
(TIF)

S12 Fig waterLOGSY spectra of GSH and analogues (all at 533 μM) in the absence (blue) and presence (green) of GFA (16 μM). Samples contained one GSH analogue (5 μL of 8 mM stock in 50 mM Tris buffer in H_2_O pH 7.5), either GFA (5 μL of 5.0 mg/mL in 50 mM Tris buffer in H_2_O pH 7.5) or 50 mM Tris buffer in H_2_O pH 7.5 (5 μL), H_2_O (61.25 μL) and D_2_O (3.75 μL). Only GSH was observed to bind GFA. Experiments were carried out at 280 K. τ_m_ = 1 s.
(TIF)

S13 Fig Bar graph showing the intensities of GSH, *N*-acetyl-cysteine, γ-Glutamyl-cysteine, *N*-acetyl-cysteamine and γ-glutamyl-D-cysteinyl-glycine (D-GSH) EXSY-correlations relative to the resonances of the corresponding *S*-hydroxymethyl HCHO adducts in the absence (blue) and presence (green) of GFA. GSH and analogues (25 μL of 40 mM stock in 50 mM Tris buffer in D_2_O pD 7.5), HCHO (25 μL of 40 mM in D_2_O) and D_2_O (20 μL) were left to pre-equilibrate before addition of His-tagged GFA (5 μL of 5.7 mg/mL in 20 mM HEPES buffer in H_2_O pH 7.5) and 1D EXSY analysis (Final volume = 75 μL). GFA only induced an increase in EXSY-correlation intensity in samples with GSH. D-GSH was at 5.3 mM. t_m_ = 80 ms. Errors are reported as standard deviations of the mean (n = 3, except for samples with D-GSH, where n = 2).
(TIF)

S14 Fig Graphs showing time-dependent formation of HMG in the absence and presence of GFA, using ^1^H NMR. Samples either contained GFA or relevant buffer (see below). The experimental conditions for each experiment were as follows: (A) GSH (25 μL of 40 mM in 50 mM BisTris buffer pD 6.0), HCHO (25 μL of 40 mM in D_2_O), His-tagged GFA (5 μL of 5.7 mg/mL in 20 mM HEPES buffer pH 7.5, or just buffer in no-enzyme control, see above), D_2_O (19 μL), and trimethylsilyl-2,2,3,3-tetradeuteropropionic acid (1 μL of 1 mg/mL in D_2_O); (B) GSH (5.8 μL of 160 mM in H_2_O, buffered to pH 6.0), HCHO (12.5 μL of 80 mM in D_2_O), D_2_O (12.5 μL), non-His-tagged GFA (5 μL of 23 mg/mL in 50 mM Tris buffer pH 7.5), and 50 mM Tris-d11 0.02% NaN_3_ buffer pD 7.5 (39.2 μL); (C) GSH (25 μL of 40 mM in 50 mM Tris-d11 buffer pD 7.5), HCHO (25 μL in D_2_O), non-His-tagged GFA (5 μL of 5 mg/mL in 50 mM Tris buffer pH 7.5), D_2_O (19 μL), and trimethylsilyl-2,2,3,3-tetradeuteropropionic acid (1 μL of 1 mg/mL in D_2_O); (D) GSH (25 μL of 2 mM in 50 mM Tris-d11 buffer pD 7.5), HCHO (1.25 μL of 40 mM in D_2_O), non-His-tagged GFA (5 μL of 5 mg/mL in 50 mM Tris buffer pH 7.5), D_2_O (42.75 μL), and trimethylsilyl-2,2,3,3-tetradeuteropropionic acid (1 μL of 1 mg/mL in D_2_O); (E) GSH (25 μL of 40 mM in 50 mM Tris buffer pH 7.5), HCHO (25 μL of 40 mM in D_2_O), non-His-tagged GFA (20 μL of 5 mg/mL GFA in 50 mM Tris buffer pH 7.5), D_2_O (4 μL), and trimethylsilyl-2,2,3,3-tetradeuteropropionic acid (1 μL of 1 mg/mL in D_2_O); (F) GSH (10 μL of 40 mM in 50 mM Tris buffer pH 7.5), HCHO (10 μL of 40 mM HCHO in D_2_O), non-His-tagged GFA (15 μL of 5 mg/mL in 50 mM Tris buffer pH 7.5), 50 mM Tris buffer pH 7.5 (5 μL), D_2_O (34 μL), and trimethylsilyl-2,2,3,3-tetradeuteropropionic acid (1 μL of 1 mg/mL in D_2_O); (G) GSH (25 μL of 4 mM in 50 mM Tris buffer pH 7.5), HCHO (2.5 μL of 40 mM in D_2_O), non-His-tagged GFA (1 μL of 5 mg/mL in 50 mM Tris buffer pH 7.5), D_2_O (45.5 μL), and trimethylsilyl-2,2,3,3-tetradeuteropropionic acid (1 μL of 1 mg/mL in D_2_O); (H) GSH (25 μL of 4 mM in 50 mM BisTris buffer pD 6.0), HCHO (2.5 μL of 4 mM HCHO in D_2_O), non-His-tagged GFA (5 μL of 5 mg/mL in 50 mM Tris buffer pH 7.5), D_2_O (41.5 μL), and trimethylsilyl-2,2,3,3-tetradeuteropropionic acid (1 μL of 1 mg/mL in D_2_O); (I) GSH (5.8 μL of 160 mM in H_2_O, buffered to pH 7.5), HCHO (25 μL of 40 mM in D_2_O), non-His-tagged GFA (23 μL of 5 mg/mL in 50 mM Tris buffer pH 7.5), and 50 mM Tris-d11 0.02% NaN_3_ buffer pD 7.5 (21.2 μL); (J) GSH (60 μL of 40 mM in 50 mM Tris-d11 buffer pH 7.5), HCHO (60 μL of 40 mM in H_2_O), non-His-tagged GFA (16 μL of 4.2 mg/mL in 50 mM Tris buffer pH 7.5), and D_2_O (24 μL). All experiments were carried out at 298 K.
(TIF)

S15 Fig ^1^H NMR spectrum of [^13^C]-HCHO. The samples contained [^13^]-HCHO (6 mM from 20 wt. % in H_2_O (Sigma Aldrich)) and Tris-d11 buffer pH 7.5 (90% H_2_O, 10% D_2_O). Resonances for hydrated [^13^C]-HCHO, [^13^C]-methanol (contaminant) and a Tris [^13^C]-HCHO adduct are highlighted. The intensities of resonances close to the solvent water resonance (δ_H_ 4.7 ppm) are reduced due to suppression (excitation sculpting). Each resonance (1–3) appears as a doublet due to the presence of one-bond coupling with ^13^C.
(TIF)

S16 Fig Addition of HCHO to *E*. *coli* cell lysate results in sequestration of endogenous GSH to form HMG. ^1^H NMR spectra of the cell lysate reveal the presence of endogenous GSH (β-cysteinyl resonance at δ_H_ = 2.87 ppm, red). The concentration of endogenous GSH decreases upon addition of HCHO with concomitant production of HMG (β-cysteinyl resonances at δ_H_ = 2.96 and 3.09 ppm, blue).
(TIF)

S17 Fig Analytic Size-Exclusion Chromatogram of wild type non-tagged GFA. Calibration with standard protein samples (black) suggest GFA (red) is a homodimer in solution (Mw = 42 kDa).
(TIF)

S18 Fig Views from a crystal structure of homodimeric GFA reveal the proximity of the potential ‘active sites’ (PDB ID: 1X6M)[14]. The monomers of the GFA homodimer interact via electrostatic and hydrophobic interactions between residues within alpha-helices, which position the partially exposed trigonal planar zinc binding sites together to form a cleft along the dimer interface. There is sufficient space within the cleft to accommodate two molecules of GSH (as observed in a crystal structure of GSH bound to GFA, PDB ID: 1XA8[14]), and, potentially, one molecule of GSH and one molecule of HMG (note HMG is unlikely to form a disulphide with the protein).
(TIF)

S1 Scheme Proposed mechanism of GFA-catalysed formation of *S*-hydroxymethylglutathione (HMG) from glutathione (GSH) and formaldehyde (HCHO), as proposed by Neculai *et al*[14]. Incubation of GFA with GSH results in loss of the crystallographically observed trigonal planar coordinated zinc(II) ion, presumably via reaction of C54 and oxidised GSH (GSSG). It is proposed that the zinc ion then binds at another protein binding site, possibly via interactions with GSH molecules as well as protein ligands. The zinc ion can then catalyse reaction of GSH and HCHO to form HMG. After the reaction, the zinc ion returns to the trigonal planar binding site.
(TIF)

S2 Scheme Reaction schemes for the initial products of reactions of GSH with HCHO, acetaldehyde and propionaldehyde. For acetaldehyde and propionaldehyde, reaction with GSH forms two diastereomeric hydroxyalkyl adducts.
(TIF)

S3 Scheme Possible metabolic pathways of HCHO in *Escherichia coli*. Upon addition of HCHO, production of formate, and at high HCHO concentration, methanol was observed. However, it is unclear which pathways of formate and methanol production predominate (note: it is also possible that other pathways producing formate and methanol from HCHO are operational).
(TIF)
