# Supplementary figures and images for: Studies on the Glutathione-Dependent Formaldehyde-Activating Enzyme from Paracoccus denitrificans
Source: PLoS One. 2015 Dec 16;10(12):e0145085. doi: 10.1371/journal.pone.0145085 (PMC4682968; doi:10.1371/journal.pone.0145085)

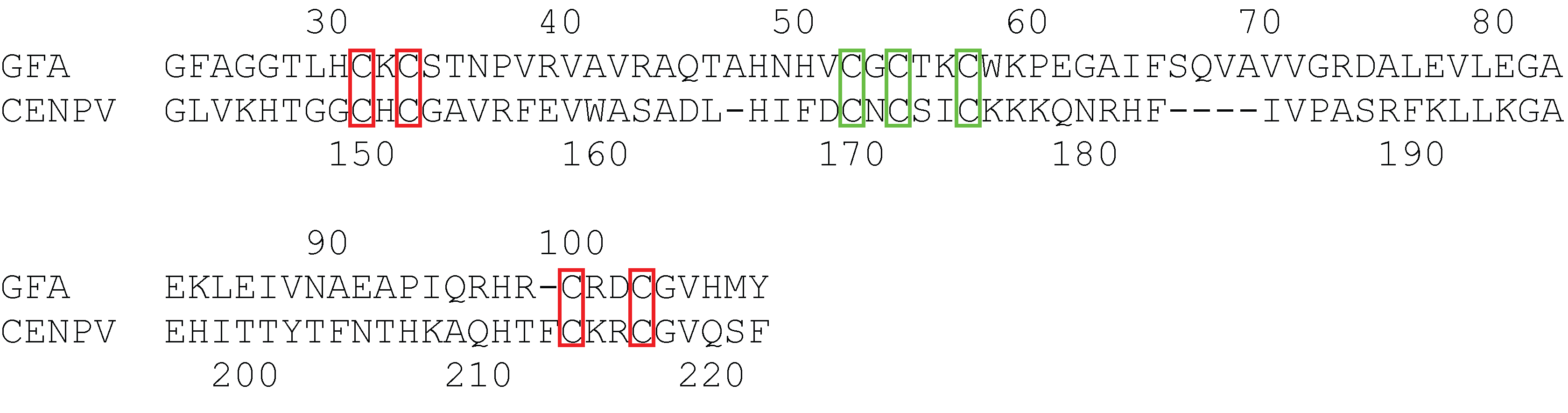

Supplement: S1 Fig — Cysteine residues predicted to coordinate zinc ions are highlighted in red (for the tetrahedral coordinated zinc ion) and green (for the trigonal planar coordinated zinc ion) respectively. In this work, full-length GFA was used (GFA WT, residues 1–194); an additional glycine residue and a histidine residue were also present on the N-terminus of this construct as a result of TEV protease-mediated removal of the N-terminal His-Tag after purification. (TIF) [file pone.0145085.s001.tif]

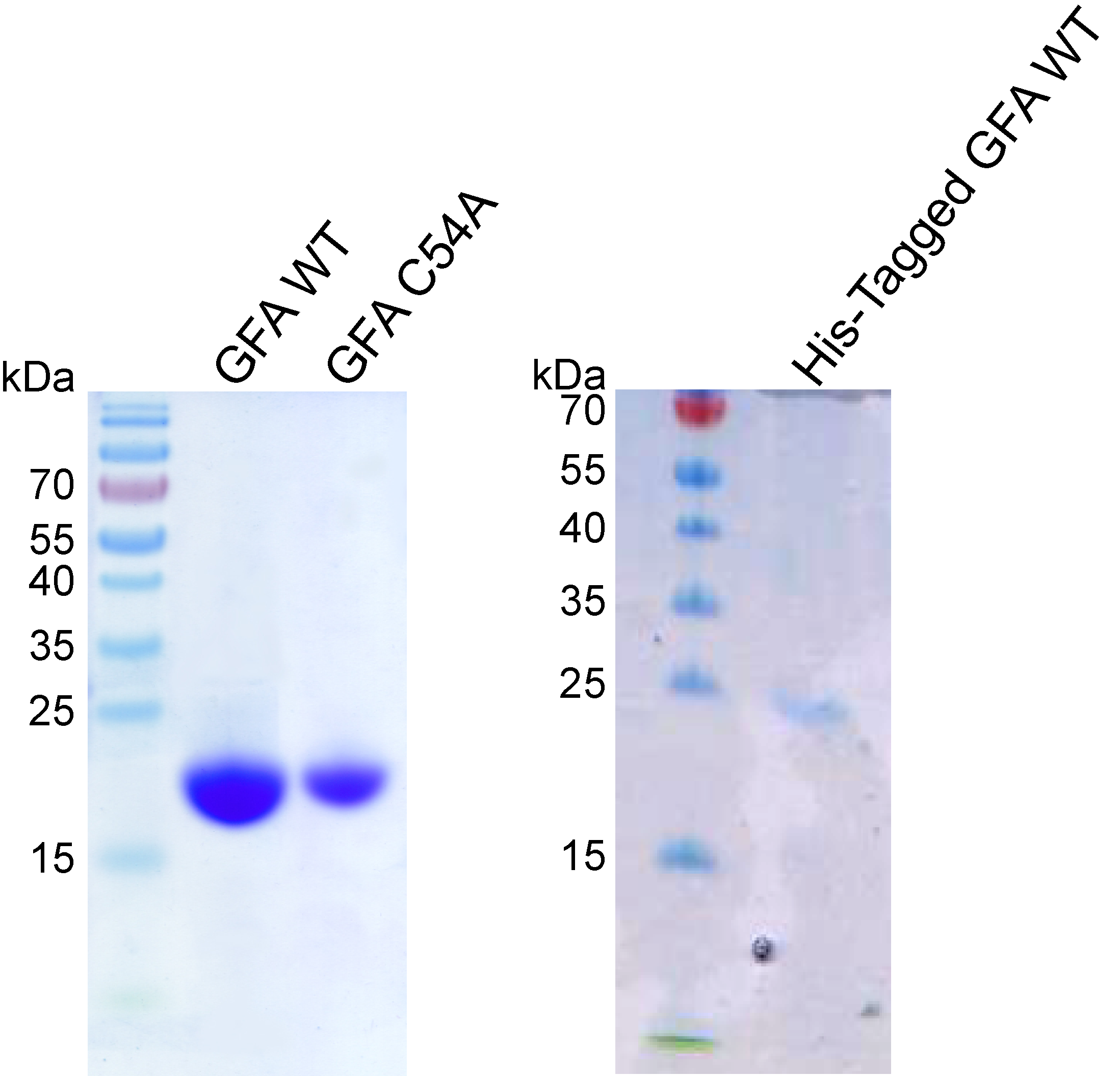

Supplement: S2 Fig — (TIF) [file pone.0145085.s002.tif]

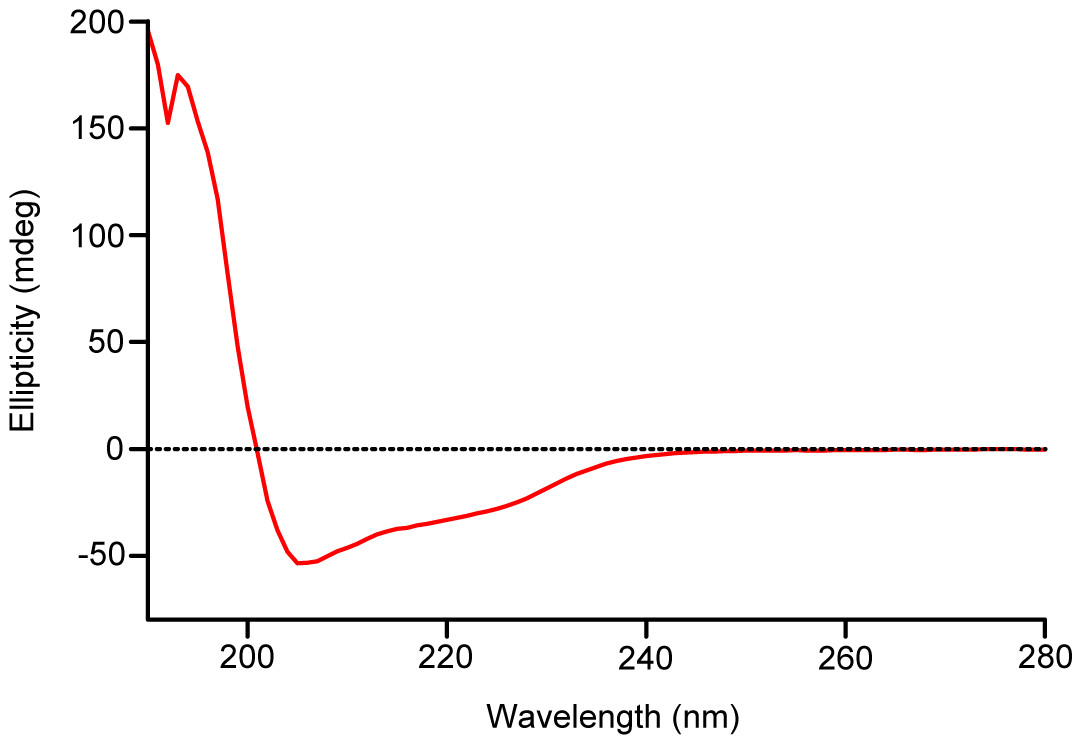

Supplement: S4 Fig — (TIF) [file pone.0145085.s004.tif]

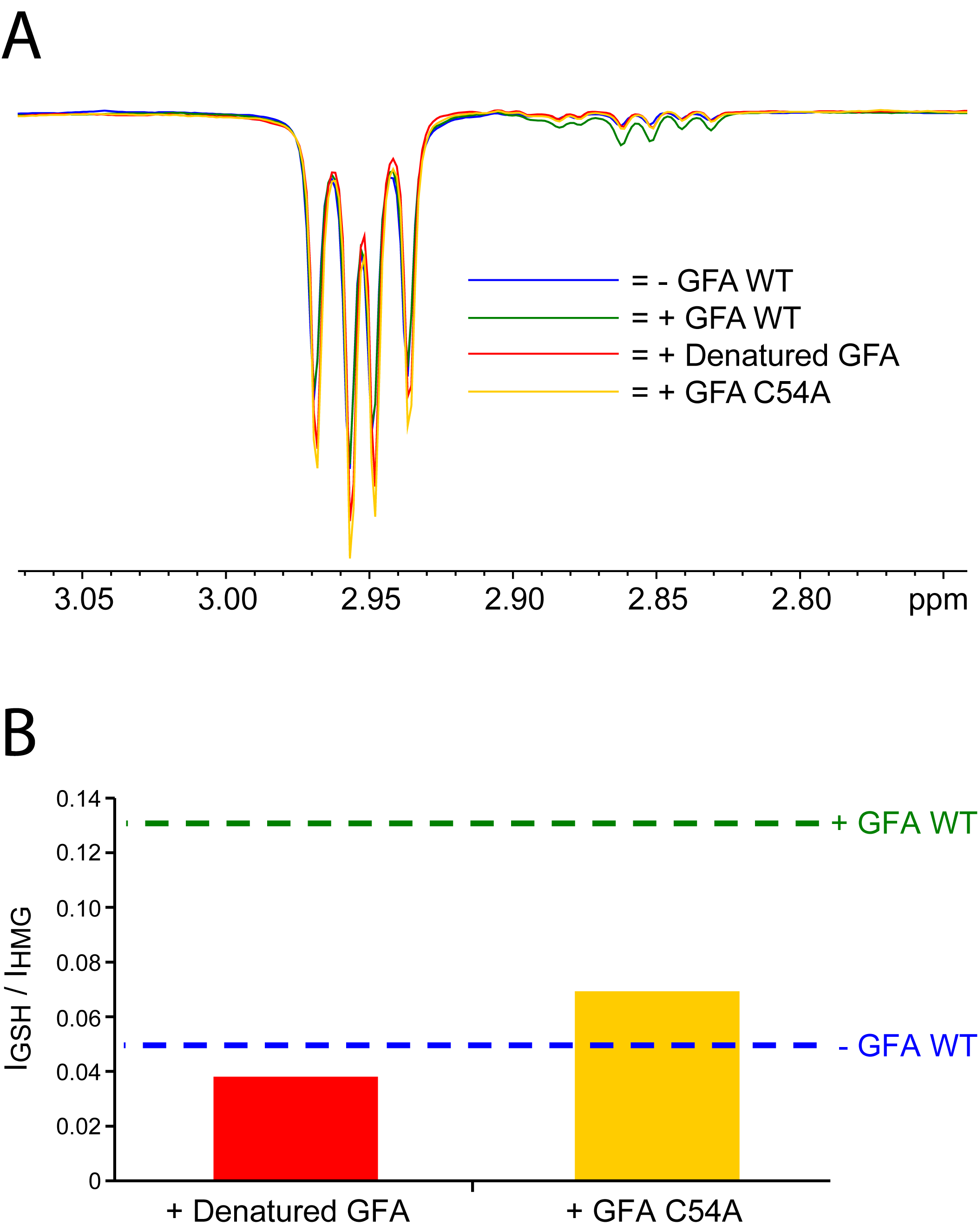

Supplement: S5 Fig — (A) 1D EXSY spectra of equilibrium mixtures of GSH (initial concentration 15 mM) and HMG in the absence (blue) and presence of GFA (20 μM, green), in the presence of GFA after denaturation (red), and in the presence of GFA C54A (20 μM, yellow). The EXSY-type correlation at δH 2.87 ppm, corresponding to the β-cysteinyl resonance of GSH, was observed to increase only in the presence of GFA (green), relative to the no enzyme control (blue). (B) Bar graph showing the intensity of the GSH EXSY-correlation relative to the inverted HMG resonance in the presence of (red) GFA after denaturation, and in the presence of (yellow) GFA C54A. Values for samples with and without wild-type GFA are indicated by dashed lines (blue and green respectively, Fig 2C). Denaturation was performed by incubating the protein in boiling water for two minutes. EXSY mixing time (τm) = 80 ms. (TIF) [file pone.0145085.s005.tif]

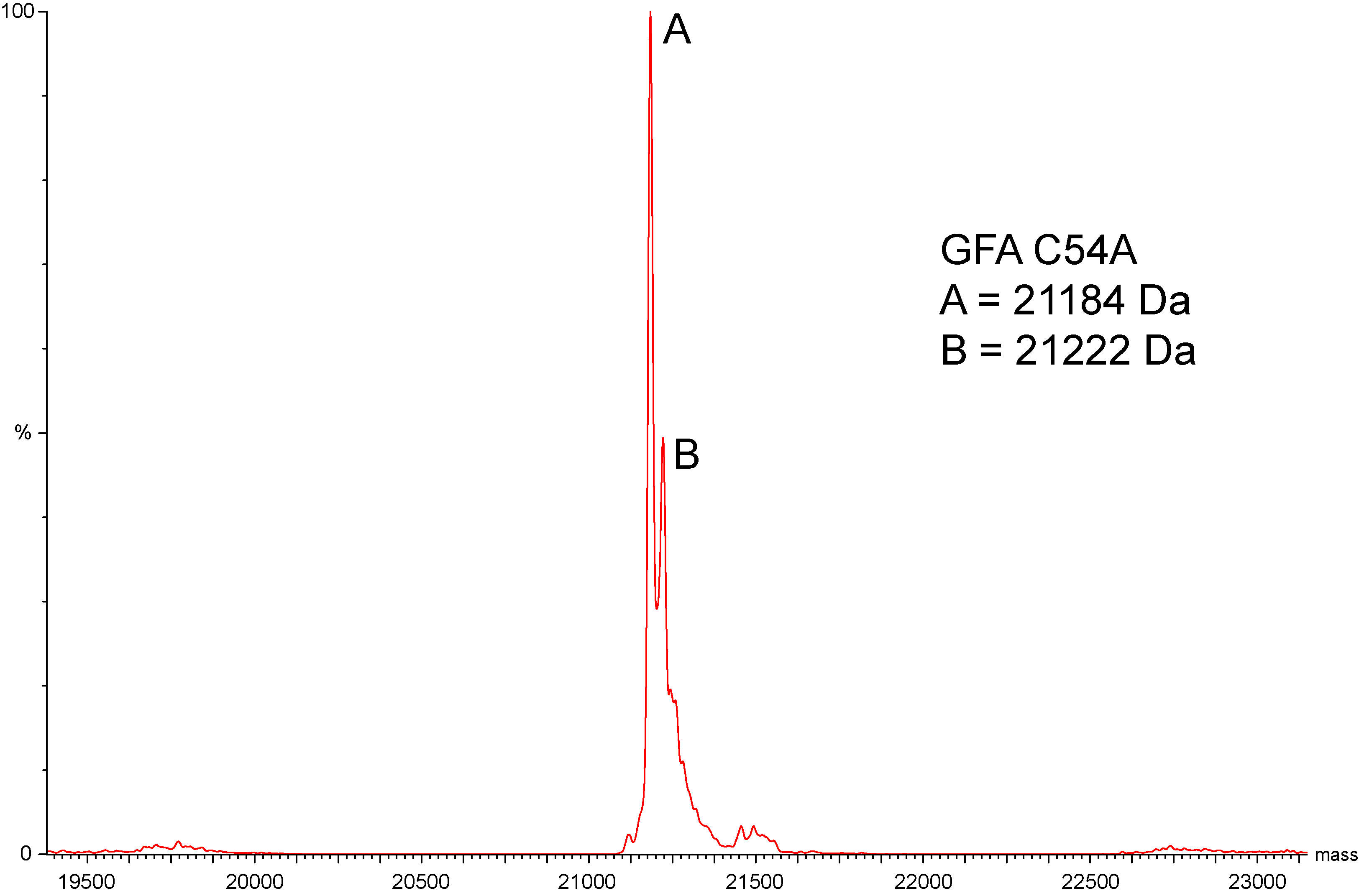

Supplement: S6 Fig — The major species (A) has a mass of 21184 Da, which is assigned to the protein in complex with one zinc ion (predicted at 21191 Da assuming deprotonation of two zinc-binding residues). The predicted mass of the protein in complex with two zinc ions is 21254 Da (assuming deperotonation of four zinc-binding residues), which is not observed. (TIF) [file pone.0145085.s006.tif]

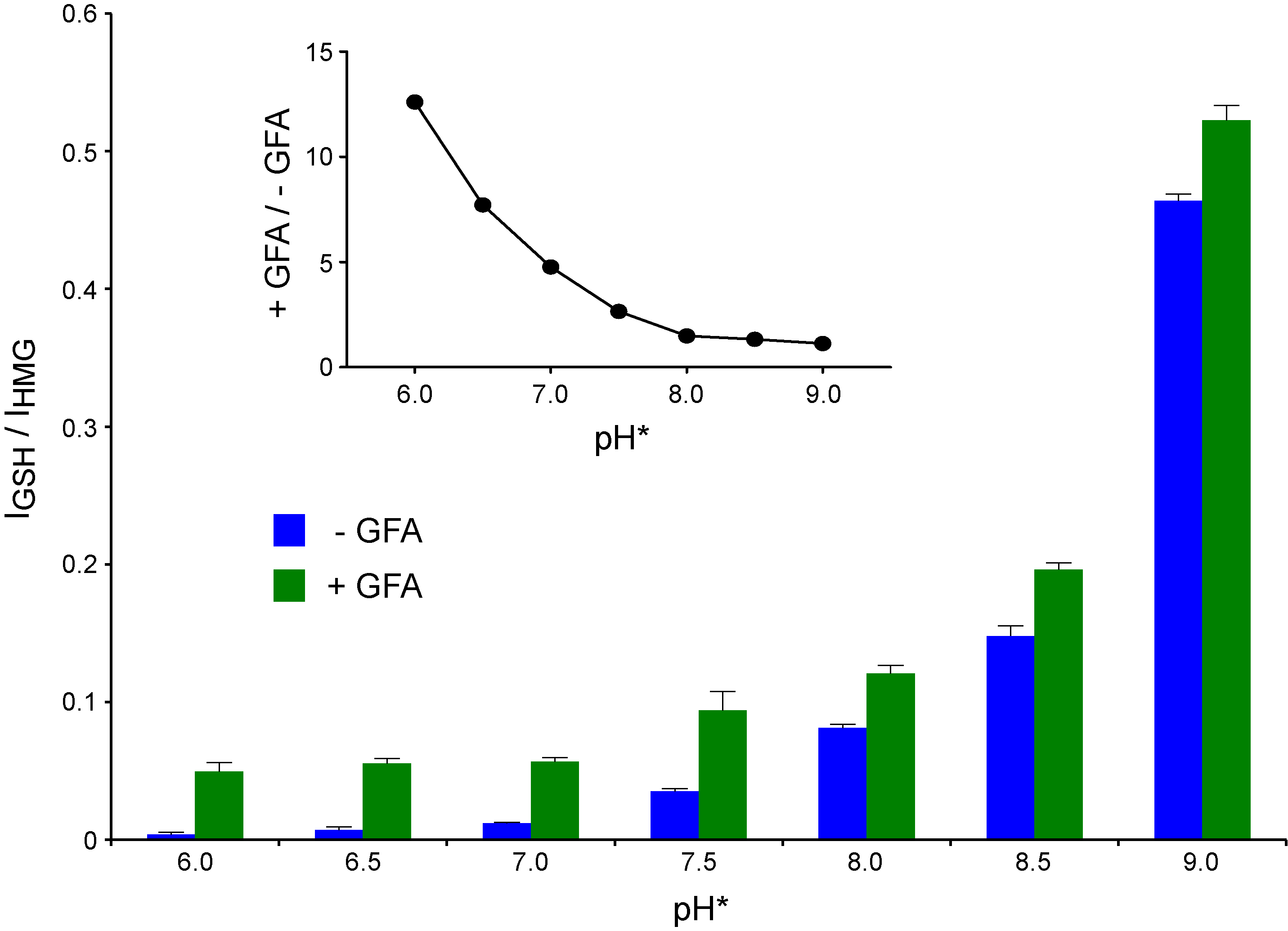

Supplement: S7 Fig — Experiments were conducted in either 50 mM Tris buffer or 50 mM BisTris buffer in D2O. GSH (25 μL of 40 mM stock in the one of the above buffers), HCHO (25 μL of 40 mM stock in D2O) and D2O (20 μL) were left to pre-equilibrate before addition of His-tagged GFA (5 μL of 5.7 mg/mL in 20 mM HEPES in H2O pH 7.5) and 1D EXSY analysis. τm = 80 ms. Inset: Graph showing the ratio of the GSH EXSY-correlation intensities in the absence and presence of GFA at different pH values. Although the EXSY correlation intensities were greater at high pH, GFA induced a greater intensity change (relative to the no enzyme control) at low pH. Errors are reported as standard deviations of the mean (n = 3, except for pH* 6.5 without GFA, where n = 2). (TIF) [file pone.0145085.s007.tif]

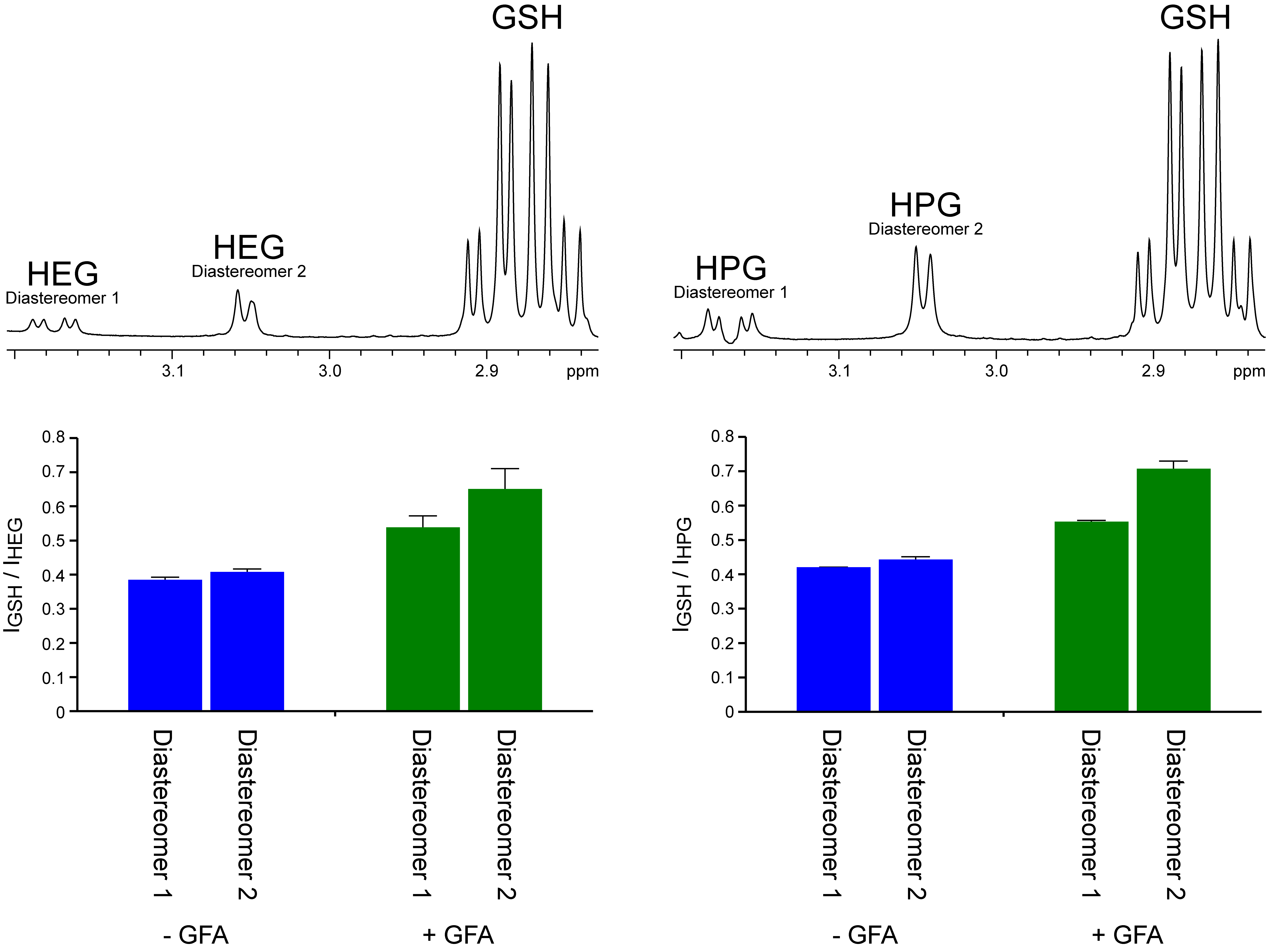

Supplement: S8 Fig — Top: 1H NMR spectra showing the formation of GSH-acetaldehyde adducts (S-hydroxyethylglutathione, HEG, left) and GSH-propionaldehyde adducts (S-hydroxypropylglutathione, HPG, right). Two diastereoisomers of each adduct are observed due to the formation of a stereogenic hemithioacetal group. Bottom: Bar graphs showing the intensities of the GSH EXSY-correlations relative to the inverted HEG resonance (left) and HPG resonance (right) in the absence (blue) and presence (green) of GFA (His-Tagged in 20 mM HEPES buffer pH 7.5, 5 μM). Experiments were conducted in 50 mM BisTris buffer pH* 6.0 in D2O, and contained GSH (13.3 mM) and either acetaldehyde (26.7 mM) or propionaldehyde (13.3 mM). τm = 80 ms. Errors are reported as standard deviations of the mean (for samples with HEG, n = 3; for samples with HPG, n = 2). (TIF) [file pone.0145085.s008.tif]

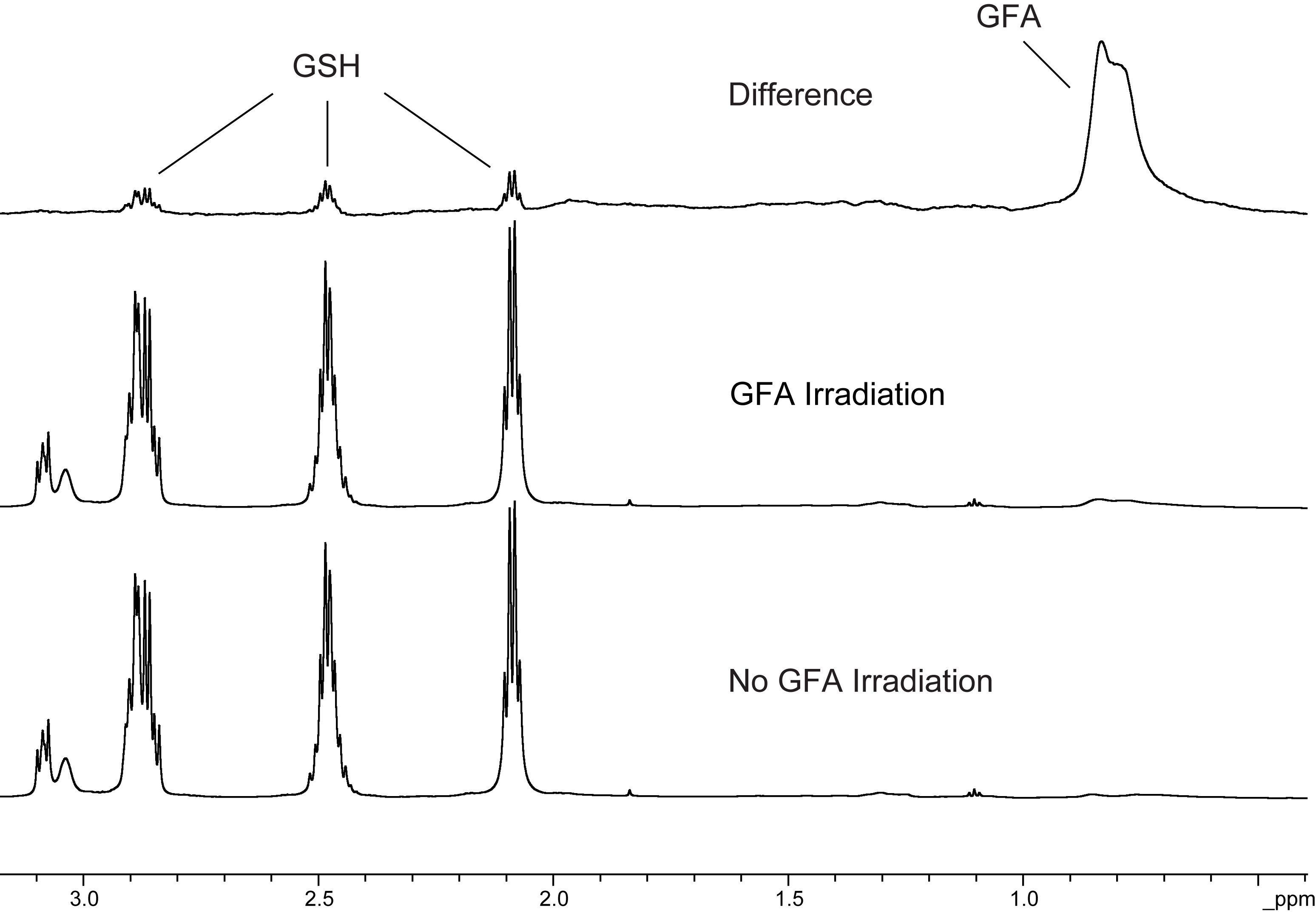

Supplement: S9 Fig — Spectra with selective irradiation at 593 Hz (corresponding to protein 1H resonances, middle) and 35000 Hz (corresponding to no 1H resonances, bottom) were collected and then subtracted to give the difference spectrum (top). The observation of 1H resonances for GSH in the difference spectrum indicates magnetisation transfer from the irradiated protein to enzyme-bound GSH (in the spectrum with irradiation at 593 Hz). The sample contained His-tagged GFA (10 μL of 5.7 mg/mL stock in 20 mM HEPES buffer in H2O pH 7.5), GSH (25 μL of 40 mM stock in Tris buffer in H2O pH 7.5) and D2O (40 μL). (TIF) [file pone.0145085.s009.tif]

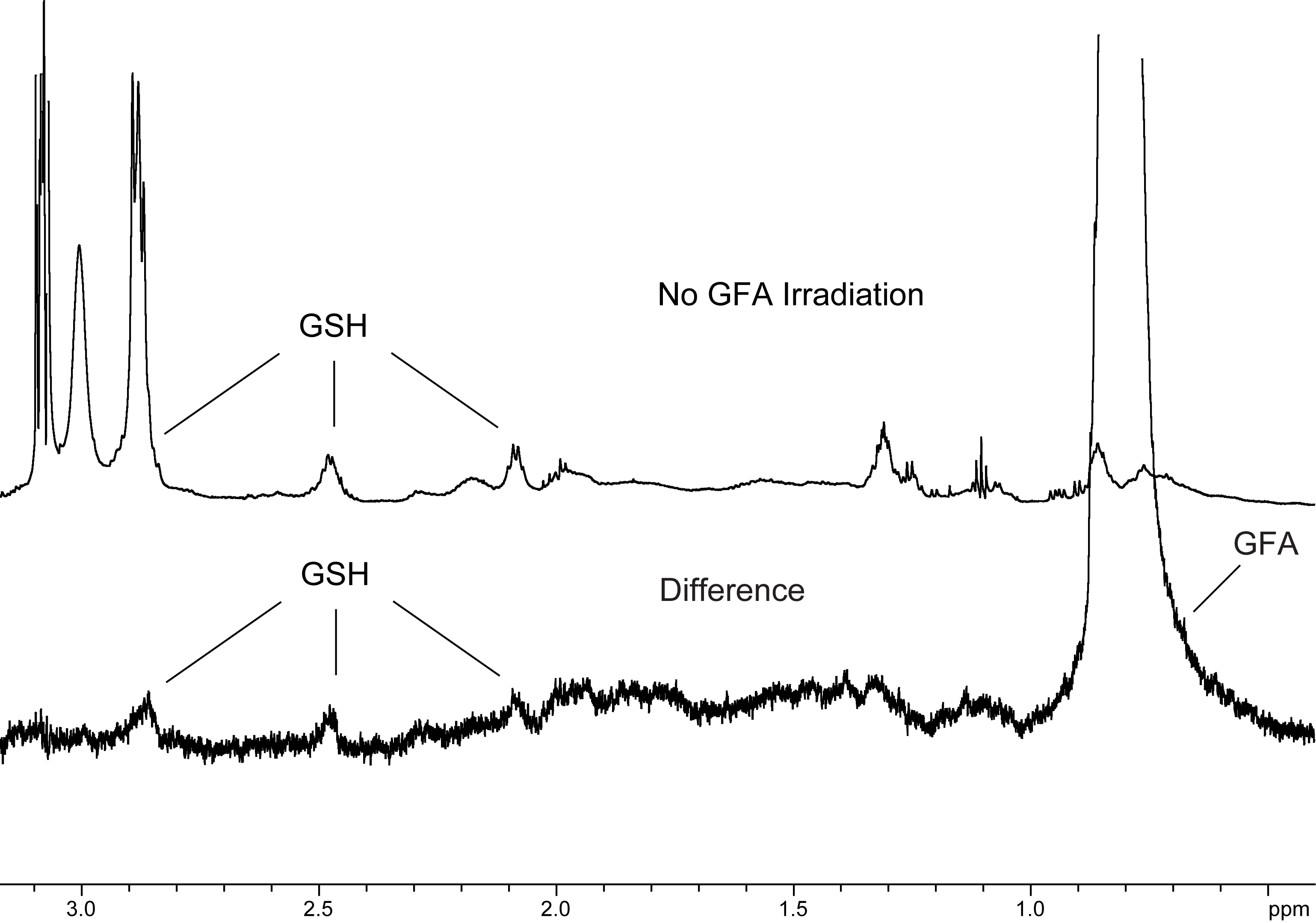

Supplement: S10 Fig — 1H resonances for GSH are observed in the difference spectrum (bottom, see S11 Fig legend for description of how the difference spectrum is obtained). The spectrum with irradiation at 35000 Hz (i.e. no protein irradiation, red), showing the GSH resonances is also shown. The sample contained His-tagged GFA (10 μL of 5.7 mg/mL stock in 20 mM HEPES buffer in H2O pH 7.5), GSH (25 μL of 1.5 mM stock in Tris buffer in H2O pH 7.5) and D2O (40 μL). (TIF) [file pone.0145085.s010.tif]

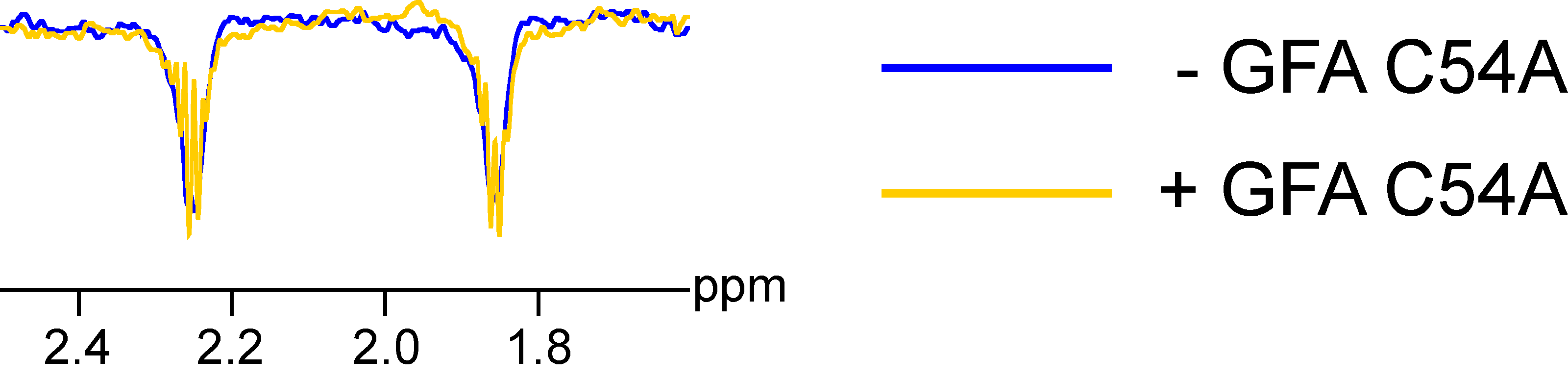

Supplement: S11 Fig — The sample contained GSH (5 μL of 8 mM stock in 50 mM Tris buffer in H2O pH 7.5), either GFA C54A (5 μL of 5.0 mg/mL in 50 mM Tris buffer in H2O pH 7.5) or 50 mM Tris buffer in H2O pH 7.5 (5 μL), H2O (61.25 μL) and D2O (3.75 μL). Experiments were carried out at 280 K. tm = 1 s. (TIF) [file pone.0145085.s011.tif]

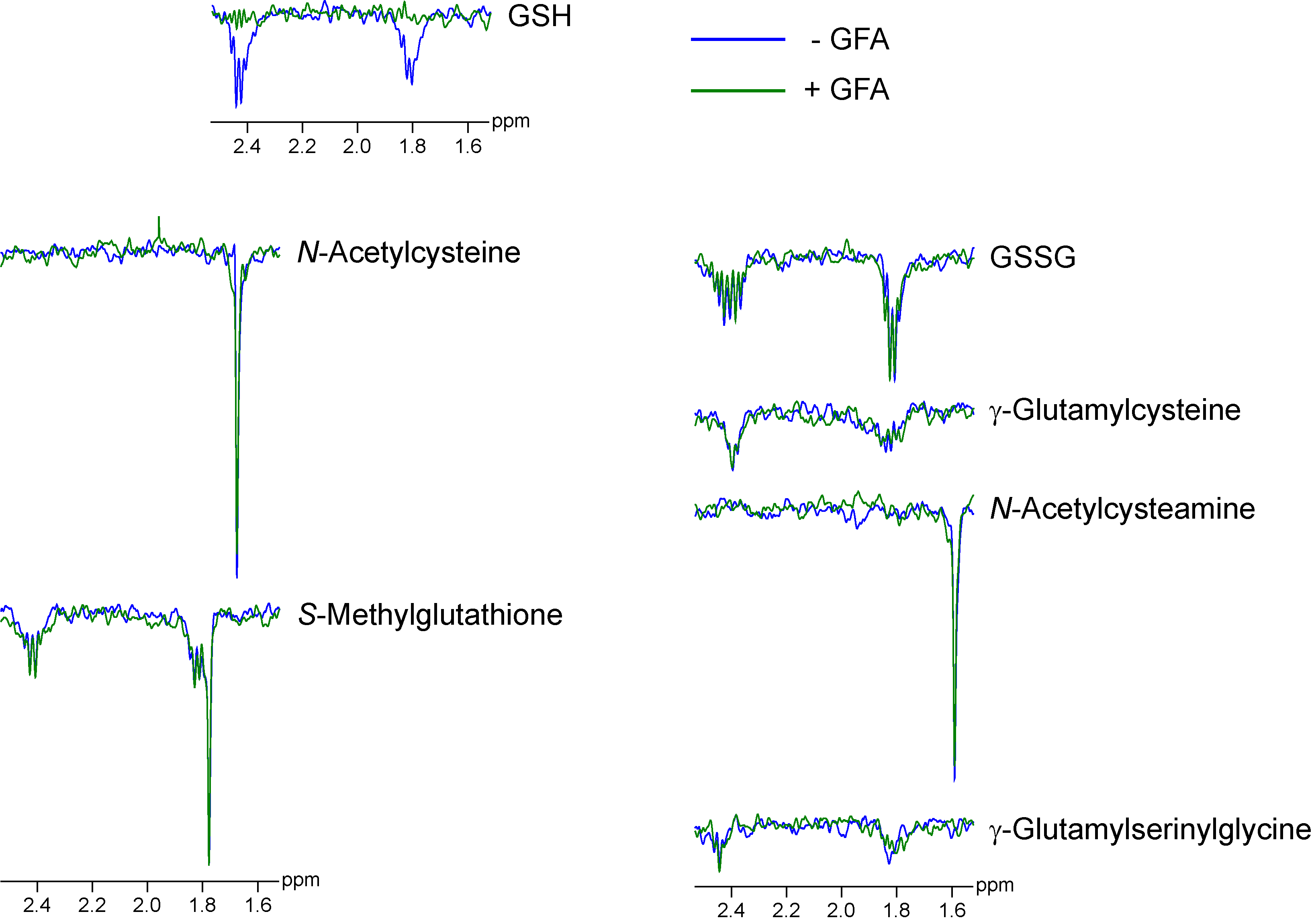

Supplement: S12 Fig — Samples contained one GSH analogue (5 μL of 8 mM stock in 50 mM Tris buffer in H2O pH 7.5), either GFA (5 μL of 5.0 mg/mL in 50 mM Tris buffer in H2O pH 7.5) or 50 mM Tris buffer in H2O pH 7.5 (5 μL), H2O (61.25 μL) and D2O (3.75 μL). Only GSH was observed to bind GFA. Experiments were carried out at 280 K. τm = 1 s. (TIF) [file pone.0145085.s012.tif]

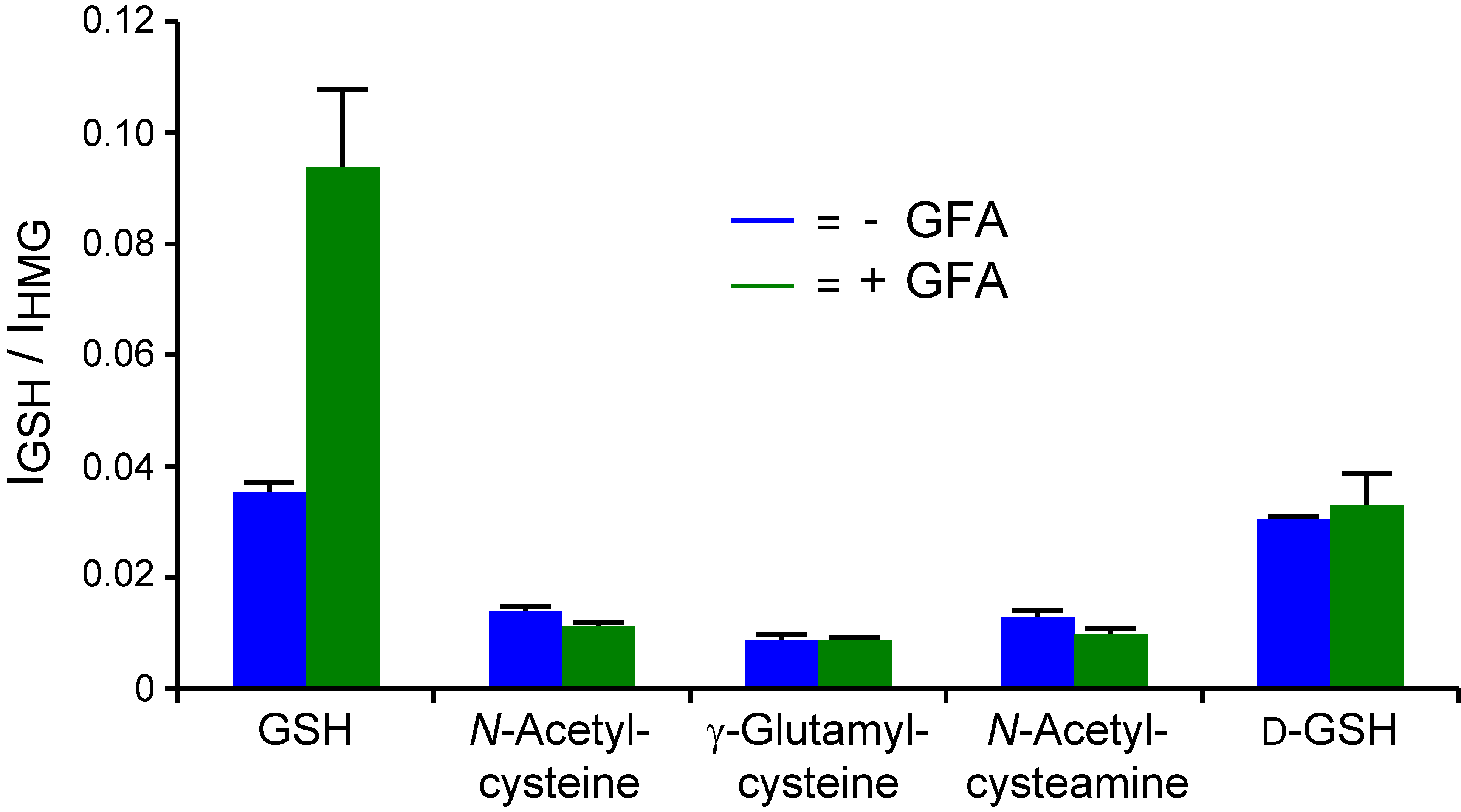

Supplement: S13 Fig — GSH and analogues (25 μL of 40 mM stock in 50 mM Tris buffer in D2O pD 7.5), HCHO (25 μL of 40 mM in D2O) and D2O (20 μL) were left to pre-equilibrate before addition of His-tagged GFA (5 μL of 5.7 mg/mL in 20 mM HEPES buffer in H2O pH 7.5) and 1D EXSY analysis (Final volume = 75 μL). GFA only induced an increase in EXSY-correlation intensity in samples with GSH. D-GSH was at 5.3 mM. tm = 80 ms. Errors are reported as standard deviations of the mean (n = 3, except for samples with D-GSH, where n = 2). (TIF) [file pone.0145085.s013.tif]

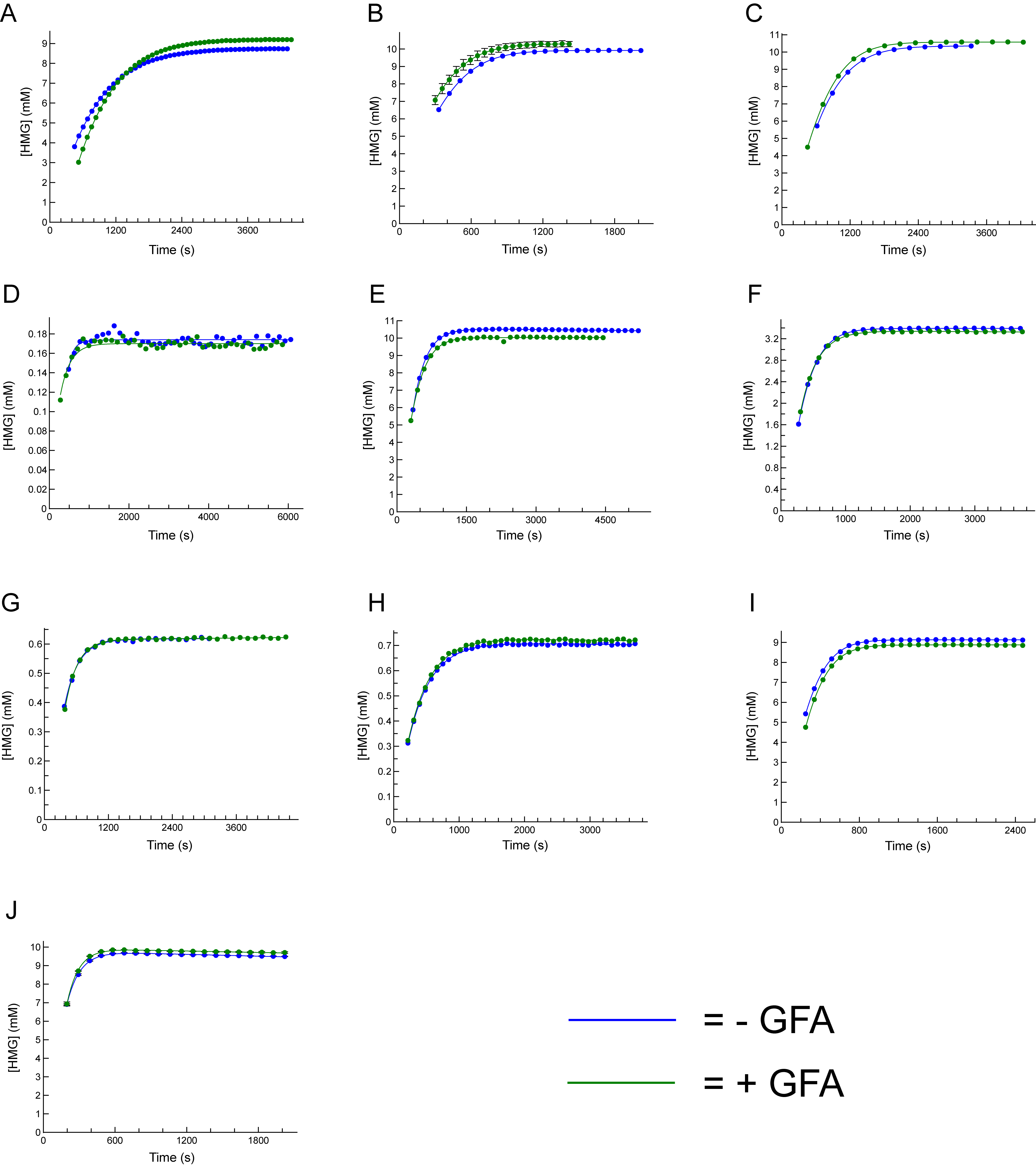

Supplement: S14 Fig — Samples either contained GFA or relevant buffer (see below). The experimental conditions for each experiment were as follows: (A) GSH (25 μL of 40 mM in 50 mM BisTris buffer pD 6.0), HCHO (25 μL of 40 mM in D2O), His-tagged GFA (5 μL of 5.7 mg/mL in 20 mM HEPES buffer pH 7.5, or just buffer in no-enzyme control, see above), D2O (19 μL), and trimethylsilyl-2,2,3,3-tetradeuteropropionic acid (1 μL of 1 mg/mL in D2O); (B) GSH (5.8 μL of 160 mM in H2O, buffered to pH 6.0), HCHO (12.5 μL of 80 mM in D2O), D2O (12.5 μL), non-His-tagged GFA (5 μL of 23 mg/mL in 50 mM Tris buffer pH 7.5), and 50 mM Tris-d11 0.02% NaN3 buffer pD 7.5 (39.2 μL); (C) GSH (25 μL of 40 mM in 50 mM Tris-d11 buffer pD 7.5), HCHO (25 μL in D2O), non-His-tagged GFA (5 μL of 5 mg/mL in 50 mM Tris buffer pH 7.5), D2O (19 μL), and trimethylsilyl-2,2,3,3-tetradeuteropropionic acid (1 μL of 1 mg/mL in D2O); (D) GSH (25 μL of 2 mM in 50 mM Tris-d11 buffer pD 7.5), HCHO (1.25 μL of 40 mM in D2O), non-His-tagged GFA (5 μL of 5 mg/mL in 50 mM Tris buffer pH 7.5), D2O (42.75 μL), and trimethylsilyl-2,2,3,3-tetradeuteropropionic acid (1 μL of 1 mg/mL in D2O); (E) GSH (25 μL of 40 mM in 50 mM Tris buffer pH 7.5), HCHO (25 μL of 40 mM in D2O), non-His-tagged GFA (20 μL of 5 mg/mL GFA in 50 mM Tris buffer pH 7.5), D2O (4 μL), and trimethylsilyl-2,2,3,3-tetradeuteropropionic acid (1 μL of 1 mg/mL in D2O); (F) GSH (10 μL of 40 mM in 50 mM Tris buffer pH 7.5), HCHO (10 μL of 40 mM HCHO in D2O), non-His-tagged GFA (15 μL of 5 mg/mL in 50 mM Tris buffer pH 7.5), 50 mM Tris buffer pH 7.5 (5 μL), D2O (34 μL), and trimethylsilyl-2,2,3,3-tetradeuteropropionic acid (1 μL of 1 mg/mL in D2O); (G) GSH (25 μL of 4 mM in 50 mM Tris buffer pH 7.5), HCHO (2.5 μL of 40 mM in D2O), non-His-tagged GFA (1 μL of 5 mg/mL in 50 mM Tris buffer pH 7.5), D2O (45.5 μL), and trimethylsilyl-2,2,3,3-tetradeuteropropionic acid (1 μL of 1 mg/mL in D2O); (H) GSH (25 μL of 4 mM in 50 mM BisTris buffer pD 6.0), HCHO (2.5 μL of 4 mM HCHO in D2O), non [file pone.0145085.s014.tif]

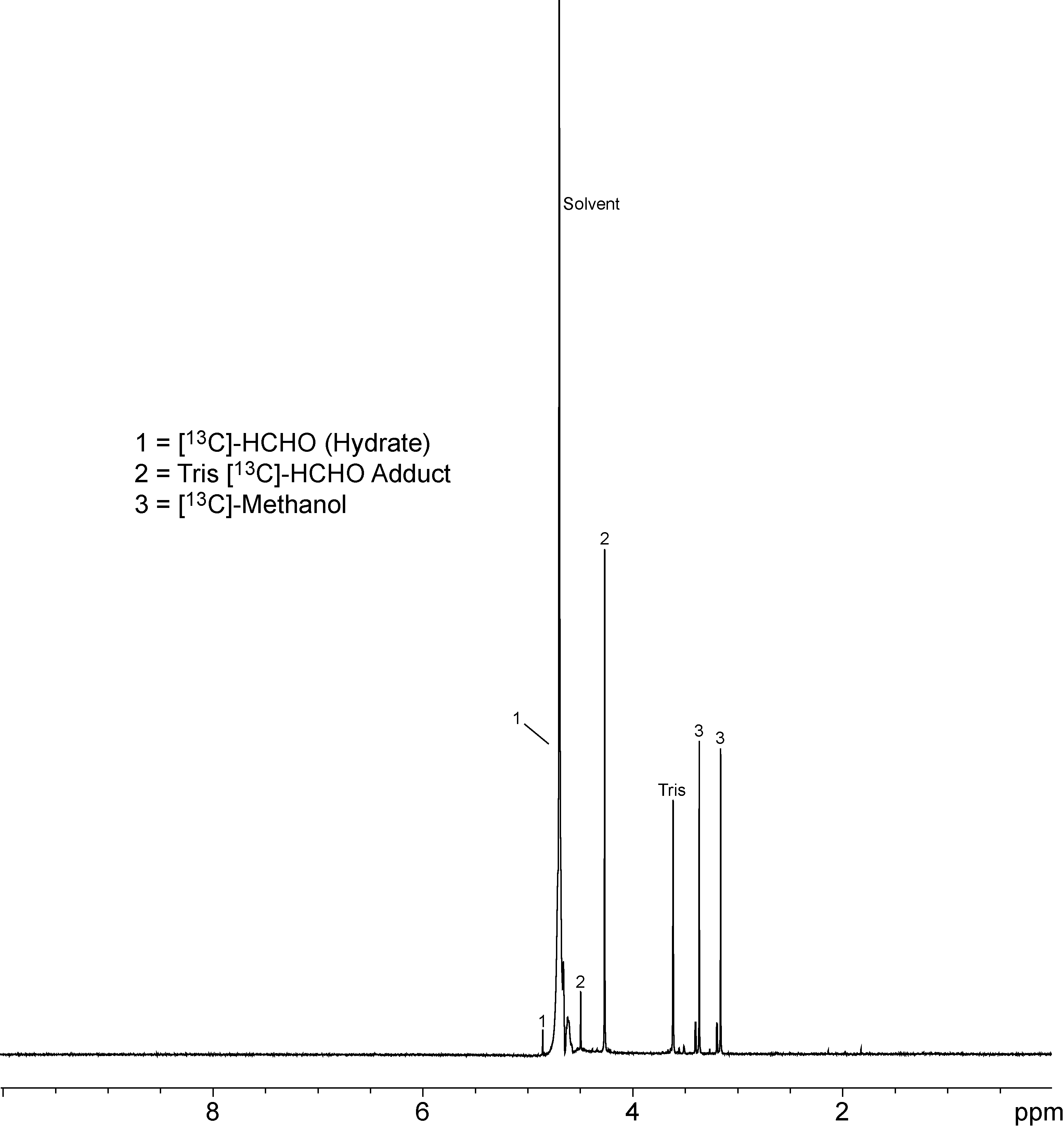

Supplement: S15 Fig — The samples contained [13]-HCHO (6 mM from 20 wt. % in H2O (Sigma Aldrich)) and Tris-d11 buffer pH 7.5 (90% H2O, 10% D2O). Resonances for hydrated [13C]-HCHO, [13C]-methanol (contaminant) and a Tris [13C]-HCHO adduct are highlighted. The intensities of resonances close to the solvent water resonance (δH 4.7 ppm) are reduced due to suppression (excitation sculpting). Each resonance (1–3) appears as a doublet due to the presence of one-bond coupling with 13C. (TIF) [file pone.0145085.s015.tif]

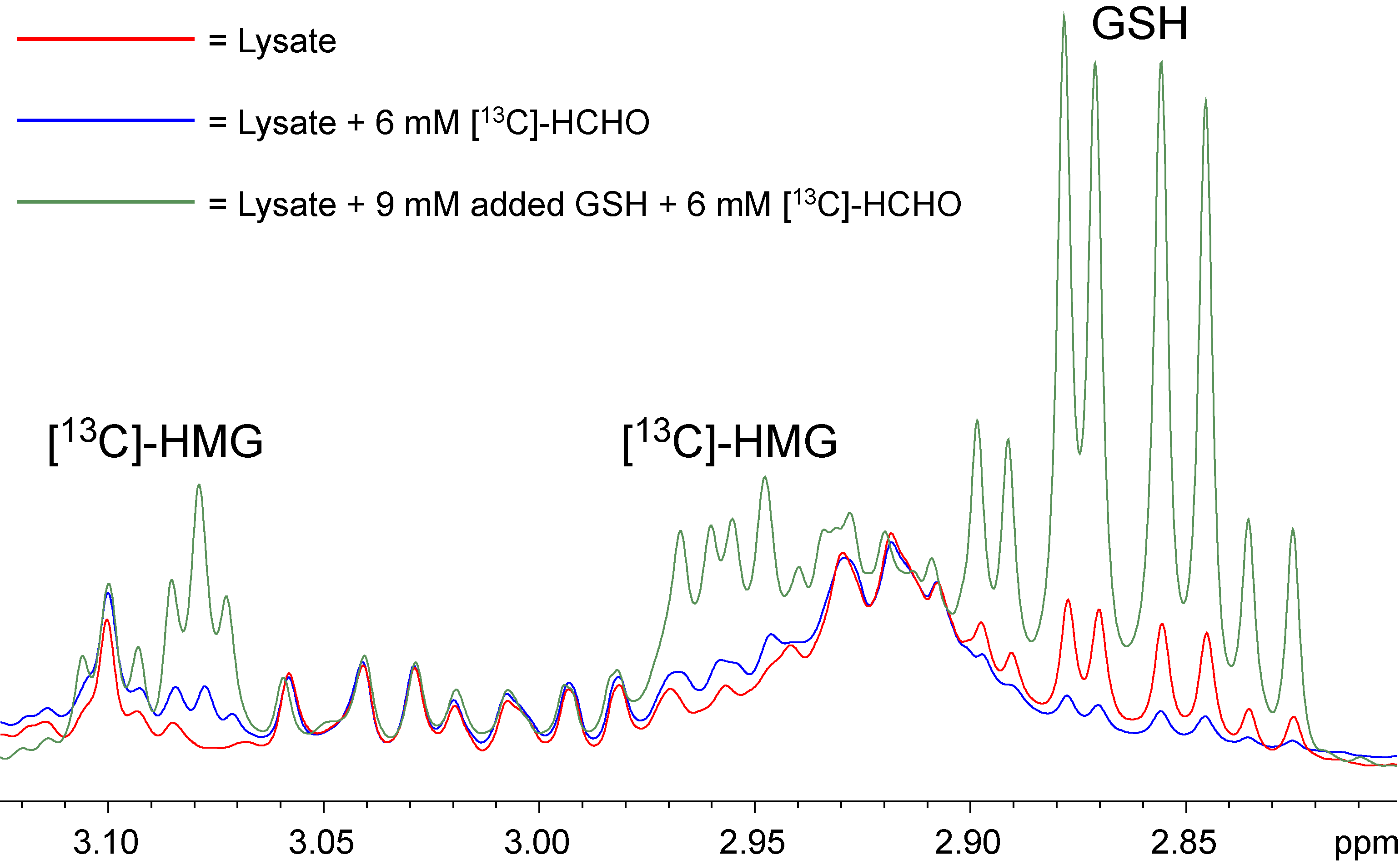

Supplement: S16 Fig — 1H NMR spectra of the cell lysate reveal the presence of endogenous GSH (β-cysteinyl resonance at δH = 2.87 ppm, red). The concentration of endogenous GSH decreases upon addition of HCHO with concomitant production of HMG (β-cysteinyl resonances at δH = 2.96 and 3.09 ppm, blue). (TIF) [file pone.0145085.s016.tif]

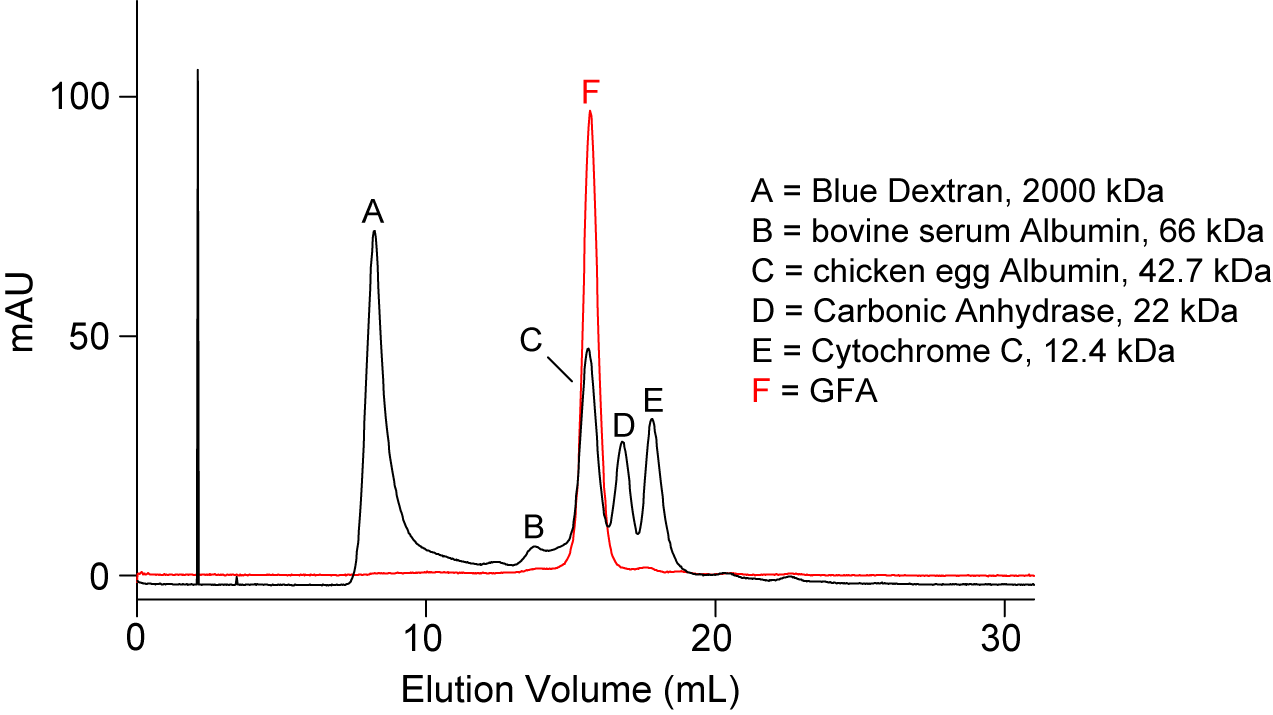

Supplement: S17 Fig — Calibration with standard protein samples (black) suggest GFA (red) is a homodimer in solution (Mw = 42 kDa). (TIF) [file pone.0145085.s017.tif]

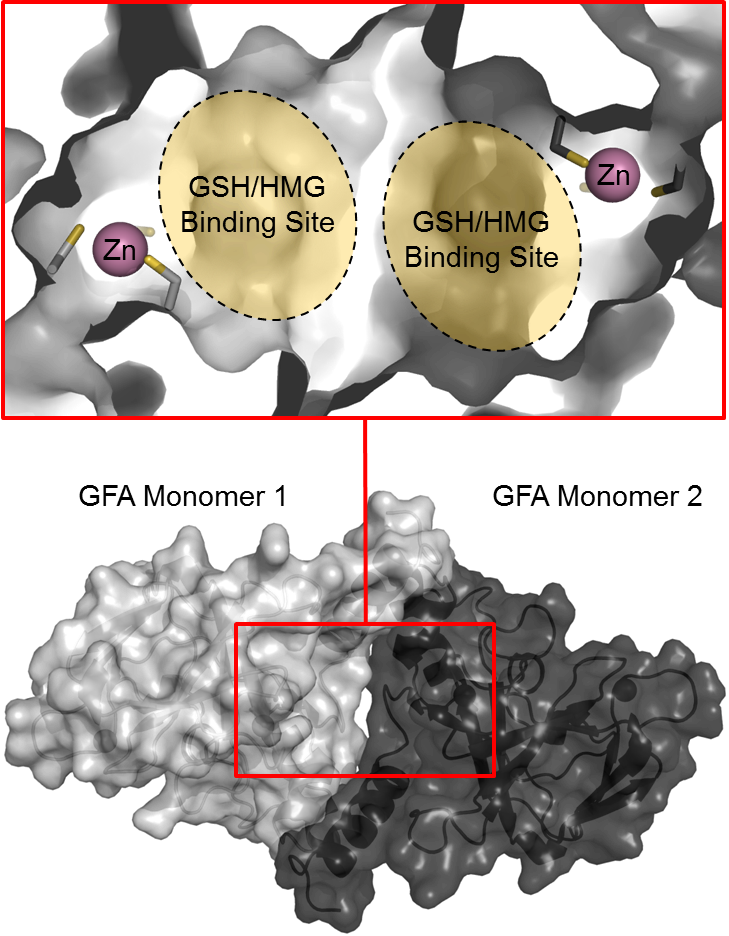

Supplement: S18 Fig — The monomers of the GFA homodimer interact via electrostatic and hydrophobic interactions between residues within alpha-helices, which position the partially exposed trigonal planar zinc binding sites together to form a cleft along the dimer interface. There is sufficient space within the cleft to accommodate two molecules of GSH (as observed in a crystal structure of GSH bound to GFA, PDB ID: 1XA8[14]), and, potentially, one molecule of GSH and one molecule of HMG (note HMG is unlikely to form a disulphide with the protein). (TIF) [file pone.0145085.s018.tif]
